# IgE-neutralizing UB-221 mAb, distinct from omalizumab and ligelizumab, exhibits CD23-mediated IgE downregulation and relieves urticaria symptoms

**DOI:** 10.1172/JCI157765

**Published:** 2022-08-01

**Authors:** Be-Sheng Kuo, Chao-Hung Li, Jiun-Bo Chen, Yu-Yu Shiung, Chia-Yu Chu, Chih-Hung Lee, Yaw-Jen Liu, Je-Hung Kuo, Cindy Hsu, Hsiao-Wen Su, Ywan-Feng Li, Annie Lai, Yueh-Feng Ho, Yi-Ning Cheng, Hong-Xuan Huang, Meng-Chung Lung, Ming-Syue Wu, Fu-Hung Yang, Chen-Han Lin, William Tseng, Jasper Yang, Chia-Yin Lin, Pei-Hua Tsai, Heng-Kwei Chang, Yi-Jen Wang, Techeng Chen, Shugene Lynn, Mei-June Liao, Chang Yi Wang

**Affiliations:** 1 United BioPharma, Inc., Hsinchu, Taiwan.; 2 UBI Asia, Hsinchu, Taiwan.; 3 United Biomedical, Inc., Hauppauge, New York, USA.; 4 Department of Dermatology, National Taiwan University Hospital and National Taiwan University College of Medicine, Taipei, Taiwan.; 5 Department of Dermatology, Kaohsiung Chang Gung Memorial Hospital and Chang Gung University College of Medicine, Kaohsiung, Taiwan.

**Keywords:** Immunology, Allergy

## Abstract

Over the last 2 decades, omalizumab is the only anti-IgE antibody that has been approved for asthma and chronic spontaneous urticaria (CSU). Ligelizumab, a higher-affinity anti-IgE mAb and the only rival viable candidate in late-stage clinical trials, showed anti-CSU efficacy superior to that of omalizumab in phase IIb but not in phase III. This report features the antigenic-functional characteristics of UB-221, an anti-IgE mAb of a newer class that is distinct from omalizumab and ligelizumab. UB-221, in free form, bound abundantly to CD23-occupied IgE and, in oligomeric mAb-IgE complex forms, freely engaged CD23, while ligelizumab reacted limitedly and omalizumab stayed inert toward CD23; these observations are consistent with UB-221 outperforming ligelizumab and omalizumab in CD23-mediated downregulation of IgE production. UB-221 bound IgE with a strong affinity to prevent FcԑRI-mediated basophil activation and degranulation, exhibiting superior IgE-neutralizing activity to that of omalizumab. UB-221 and ligelizumab bound cellular IgE and effectively neutralized IgE in sera of patients with atopic dermatitis with equal strength, while omalizumab lagged behind. A single UB-221 dose administered to cynomolgus macaques and human IgE (ε, κ)–knockin mice could induce rapid, pronounced serum-IgE reduction. A single UB-221 dose administered to patients with CSU in a first-in-human trial exhibited durable disease symptom relief in parallel with a rapid reduction in serum free-IgE level.

## Introduction

There remains an unmet medical need for a diverse array of allergic diseases, of which the combined disease prevalence has substantially increased to affect more than 30% of the world’s population, causing global health threats and mounting economic burden (1, 2). Allergic (atopic) diseases such as food allergy, atopic dermatitis (AD), asthma, and allergic rhinitis can be interrelated and are sometimes referred to as “atopic march” (3–5), which may be initiated from early childhood and is largely mediated by IgE. The functions of IgE hinge on interactions of its Fc region (Cε2–Cε4) with 2 principal receptors: FcεRI, expressed mainly on mast cells and basophils and responsible for allergic hypersensitivity and inflammation; and CD23 (FcεRII), expressed mainly on B cells and involved in the regulation of IgE synthesis, IgE clearance, and a host of other immunological functions (6–9).

IgE binds with high affinity (*K_D_*, ~10^–10^–10^–11^ M) to FcԑRI (10) at sites on the Cε3 domain to form an “open” conformation (11, 12), while it binds with low affinity (*K_D_*, ~10^–6^–10^–7^ M) to a single chain (monomeric) (13, 14) of the CD23 receptor around the juncture of the Cε3 and Cε4 domains to form a “closed” conformation (15–17). Since the sites on IgE that bind to FcεRI are distant from those that bind to CD23, the 2 IgE-binding events are mutually exclusive by reciprocal allosteric inhibition, such that FcԑRI and CD23 can function independently without simultaneous engagement with IgE (9, 16–18). CD23 on the cellular surface commonly exists as a homotrimer. The IgE binding affinity for the free trimeric CD23, or interaction in the form of IgE immune complexes with CD23, could yield an avidity strength (*K_D_*, 10^–9^–10^–10^ M) approaching that of the IgE-FcԑRI interaction (8, 19, 20).

Given its limited effects against worms and cancer (21, 22), IgE, due to its harmful effector functions manifested in allergic symptoms, is generally perceived as physiologically dispensable and as a legitimate, safe target for drug development. Omalizumab binds IgE allosterically (23), preventing it from interacting with FcεRI, reducing serum free-IgE levels and subsequently downregulating FcεRI expression (24–26), thereby desensitizing effector cells. Omalizumab does not bind to the FcεRI-bound IgE, and so does not cross-link with IgE to trigger hypersensitivity like IgE-specific allergens do. Omalizumab does not bind, either, to the CD23-bound IgE (IgE-CD23 complex), as it blocks the IgE binding to CD23 orthosterically (23, 27), and as such omalizumab theoretically would fall short of playing a direct role in CD23-regulated functional activities (28, 29).

Omalizumab is the only anti-IgE antibody approved to date and is restricted to serve as a third-line add-on therapeutic for moderate-to-severe persistent allergic asthma (approved 2003), chronic spontaneous urticaria (CSU) (approved 2014), and nasal polyps (approved 2020) (30). New anti-IgE biologicals have been pursued preclinically and clinically (31, 32). However, among alternative IgE-targeting antibodies (anti-Cε) explored and remaining viable in late-phase clinical trials, the higher-affinity ligelizumab (QGE031) (33, 34) is the only candidate developed to overcome some of omalizumab’s limitations, with better inhibition of IgE binding to FcεRI and a presumably greater reduction in IgE synthesis, likely due to a limited engagement with the CD23-bound IgE despite minor binding site overlap on IgE (35). However, while ligelizumab showed anti-CSU efficacy superior to that of omalizumab in phase II (34), it did not excel in the phase III trial (https://www.hcplive.com/view/phase-3-ligelizumab-not-superior-omalizumab).

A new class of anti-IgE mAb, 8D6 (the murine mAb of the humanized UB-221), was found not to interfere with IgE binding to CD23 (36, 37), for which the binding mechanisms and functional consequences remain to be elucidated. In this report, we feature the uniqueness of UB-221 that differentiates it from omalizumab and ligelizumab. Free UB-221 binds to CD23-bound IgE and engages CD23 in an unrestricted manner when in multiple mAb-IgE complex forms, while both ligelizumab and omalizumab are much more limited in their indirect binding with CD23. The differential CD23-interaction profiles correlate with the finding that UB-221 downregulates CD23-mediated IgE neosynthesis in human PBMCs under costimulation by IL-4 and an anti-CD40 antibody.

Moreover, UB-221 binds IgE with a higher affinity than omalizumab and is superior in IgE neutralization and prevention of basophil degranulation. UB-221 and ligelizumab neutralize high serum IgE of patients with AD with equal strength, while omalizumab is less effective. In cynomolgus macaques and human IgE (ε, κ)–transgenic (hIGHE-knockin) mice, a single dose of UB-221 can induce a rapid, pronounced reduction in serum IgE. In addition, UB-221 in a phase I single-dose clinical trial involving patients with CSU (ClinicalTrials.gov NCT03632291)has demonstrated durable disease symptom relief (reduction in weekly urticaria activity score, UAS7) that associates with a rapid reduction in serum free-IgE level.

## Results

### Prevention of basophil activation and degranulation.

8D6, an anti-IgE IgG_1_ mAb, was previously reported to neutralize IgE and bind to CD23-bound IgE (36, 37). As a first step toward generating an anti-IgE therapeutic agent suitable for the treatment of IgE-mediated diseases, we humanized 8D6. The human acceptor framework used for the humanization of 8D6 was based on human germline VH1-69 and Vκ1-39 domains (Supplemental Figure 1; supplemental material available online with this article; https://doi.org/10.1172/JCI157765DS1). The humanized 8D6 was designated as UB-221. As with the prerequisite for omalizumab to be safe for therapeutic use, UB-221 does not bind to the FcεRI-bound IgE on ELISA (Supplemental Figure 2).

**Figure 1:**
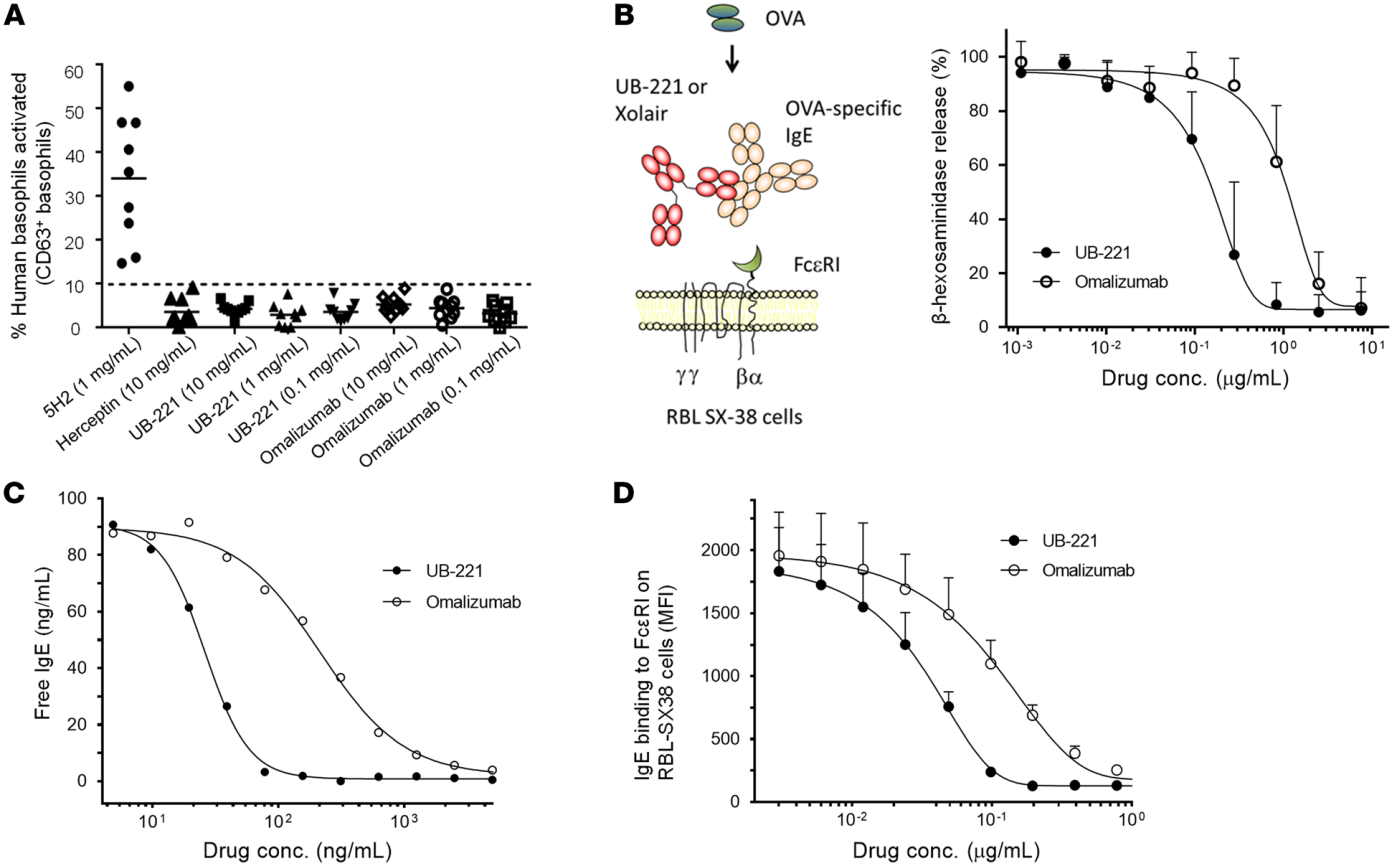
Competitive inhibition against basophil degranulation and IgE-FcεRI interactions. (**A**) As determined by CD63 expression on flow cytometry as a basophil activation marker, UB-221, omalizumab, and negative control (anti-Her2 trastuzumab mAb) do not activate isolated primary human basophils, while the positive control anti-IgE 5H2 antibody does. (**B**) In inhibition of FcԑRI-expressing RBL SX-38 cell degranulation induced by the ovalbumin-IgE (OVA-IgE) complex (mean ± SD, *n =* 6), UB-221 exhibits 7-fold greater inhibition over omalizumab (IC_50_: 0.14 vs. 0.94 μg/mL). The results are in line with higher potency in competitive inhibition of IgE-FcεRI interactions shown on ELISA and RBL SX-38 cells. (**C**) In a representative competitive inhibition assay on ELISA coated with FcεRI, UB-221 inhibits IgE binding with 8-fold greater potency over omalizumab (IC_50_: 26.1 vs. 214 ng/mL). (**D**) In competitive inhibition of IgE binding to FcεRI-expressing RBL SX-38 cells (mean ± SD, *n =* 3), UB-221 inhibits IgE binding with 3-fold greater potency over omalizumab (IC_50_: 0.035 vs. 0.106 μg/mL).

On FcεRI-expressing RBL SX-38 cells, UB-221 and omalizumab in free form or in preformed IgE-mAb complexes did not bind to the cells (Supplemental Figure 3, A and C), and so not do not trigger basophil degranulation expressed as the release of β-hexosaminidase (Supplemental Figure 3, B and D). RBL SX-38 is a rat basophilic leukemia cell line expressing the α, β, and γ chains of human FcεRI that serves as a sensitive model for exploring functional IgE-allergen interactions (38). Similarly, neither UB-221 nor omalizumab could activate human primary basophils, as indicated by the expression of the activation marker CD63 (Figure 1A).

Nevertheless, in the presence of ovalbumin (OVA) allergen and OVA-specific IgE, we observed that, as compared with omalizumab, UB-221 competed favorably to inhibit the IgE-OVA allergen complex–induced RBL SX-38 degranulation with 7-fold greater efficiency (Figure 1B), as indicated by an IC_50_ value of 0.14 versus 0.94 μg/mL, respectively. This observation is in line with an 8-fold competition edge in inhibition of IgE binding to FcεRI immobilized on ELISA, with an EC_50_ value of 26.1 versus 214 ng/mL (Figure 1C), and a 3-fold higher competitive inhibition against IgE binding to the RBL SX-38 cells, with an EC_50_ value of 35 versus 106 ng/mL (Figure 1D).

### Unrestricted binding in free form to CD23-bound IgE and in the mAb-IgE complex form to CD23.

As CD23 is involved in negative feedback regulation of IgE production (39–41), it is of interest to explore the binding capability of an anti-IgE mAb toward CD23. We observed that free UB-221 could bind to IgE preabsorbed to trimeric CD23 immobilized on ELISA (Supplemental Figure 4A), and IgE-complexed UB-221 could bind to CD23^+^ SKW6.4 B lymphoma cells (Supplemental Figure 4B), while omalizumab did not react in either binding event. This distinctive feature of UB-221 warrants its further comparison with ligelizumab.

**Figure 2:**
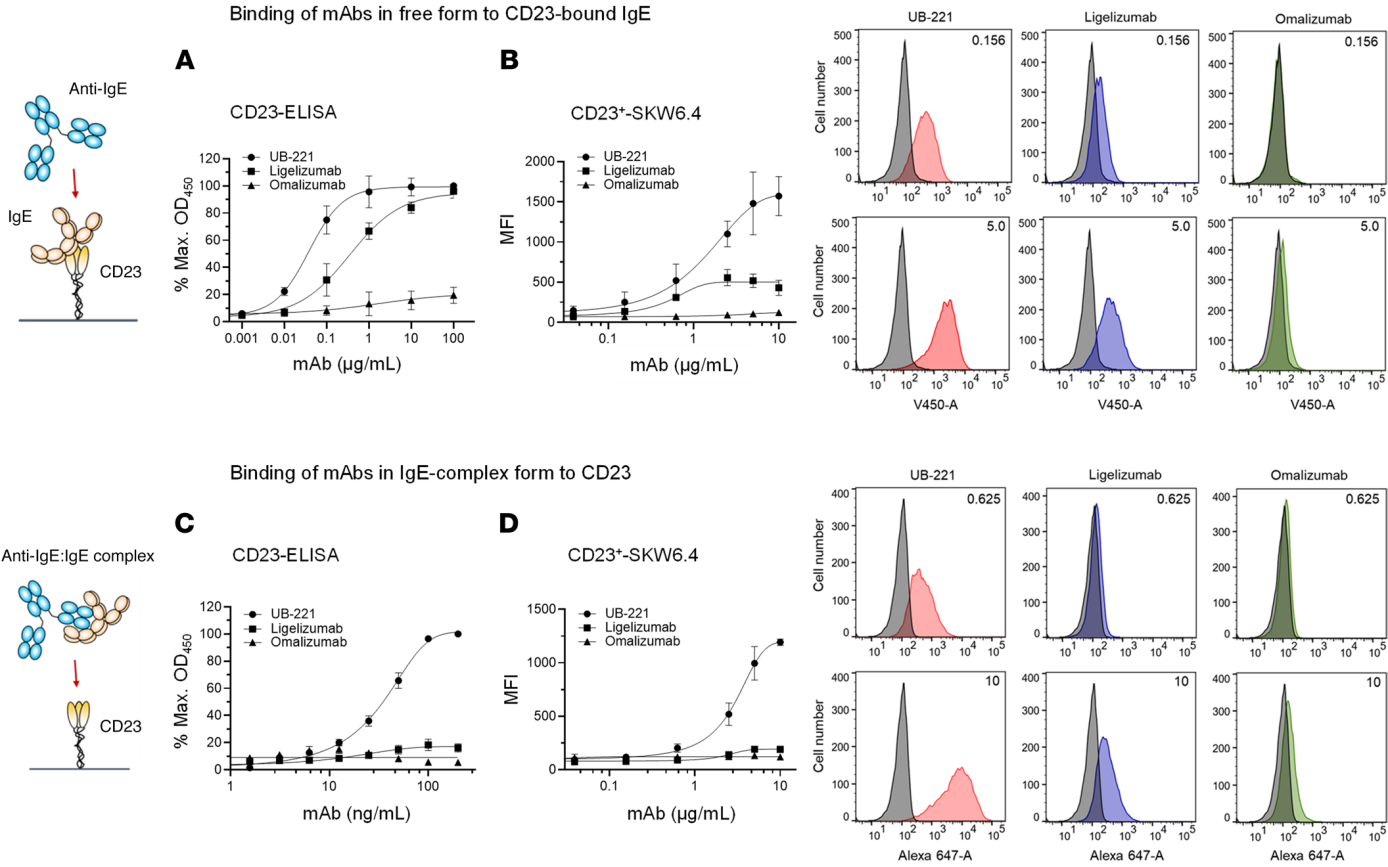
Interactions of anti-IgE mAbs in free or IgE-complex form with CD23. Anti-IgE mAbs, in free form or in mAb-IgE complex form, may interact with CD23 in an indirect fashion. (**A**) On CD23-immobilized ELISA with IgE preloaded, UB-221 in a free form exhibited strong binding to the CD23-bound IgE, estimated to be 10-fold more abundant than ligelizumab, as shown with EC_50_ values (mean ± SD, *n =* 3) of 38.4 ± 3.6 versus 402 ± 47.3 ng/mL, while omalizumab was relatively inactive. (**B**) On CD23^+^ SKW6.4 B lymphoma cells with IgE preabsorbed and analyzed by flow cytometry, UB-221 in a free form exhibited again strong binding to the CD23-bound IgE, with a maximum binding MFI value (mean ± SD, *n =* 3) of 1,828 ± 331, while ligelizumab presented an approximately 4-fold lower binding, with a maximal MFI of 503 ± 26.6, and omalizumab remained inactive; the differential cellular binding events are shown on FACS histograms on the right exemplified by 2 mAb concentrations at 0.156 and 5.0 mg/mL. (**C**) On CD23-immobilized ELISA, UB-221 in preformed UB-221–IgE complexes also exhibited strong binding to CD23, with an EC_50_ of 41.6 ± 4.6 ng/mL, nearly the same as that observed with CD23-bound IgE, while ligelizumab-IgE complexes almost lost the ability to bind CD23, and the omalizumab-IgE complexes stayed inert toward CD23. (**D**) On CD23^+^ SKW6.4 cells, the preformed UB-221–IgE complex again exhibited strong binding to the cells, with a maximal MFI value (mean ± SD, *n =* 3) of 1,280 ± 90.2, while ligelizumab almost lost the binding ability, presenting only a minor binding with a maximal MFI of 191 ± 8.5, and omalizumab was inactive; the differential cellular binding events are shown on FACS histograms on the right exemplified by 2 mAb concentrations at 0.625 and 10 μg/mL. Overall, UB-221 in free or complex form can interact freely with CD23, while ligelizumab in free form can bind limitedly to CD23-bound IgE and in IgE-complex form loses its binding capability, and omalizumab essentially stays inert toward CD23.

On CD23-immobilized ELISA with IgE preloaded, free UB-221 exhibited strong binding to the CD23-bound IgE (Figure 2A), approximately 10-fold more abundant than that by ligelizumab, as shown in the mean EC_50_ value of 39.1 versus 396 ng/mL, while omalizumab was inactive. A preformed UB-221–IgE complex bound strongly as well to CD23 (Figure 2C), with an EC_50_ of 41.6 ng/mL that is nearly the same as that exhibited in binding to the CD23-bound IgE, while ligelizumab in IgE-complex form lost its ability to bind CD23, and the omalizumab-IgE complex remained inert toward CD23.

A similar landscape of CD23 binding was evident with SKW6.4 cells, where free UB-221 avidly engaged CD23-bound IgE (Figure 2B), approximately 4-fold greater than that by ligelizumab, as shown in the maximum mean fluorescence intensity (MFI) value of 1,828 versus 503, whereas omalizumab remained inactive. The differential dose-dependent cellular binding events could be visualized through the shifting of histograms on flow cytometry, exemplified by 2 levels at 0.156 and 5.0 μg/mL.

UB-221 in IgE-immune complex form, similar to that observed in the CD23 ELISA (Figure 2C), could bind freely to CD23^+^ SKW6.4 cells (Figure 2D), while ligelizumab lost the CD23 binding capability. Again, omalizumab in IgE-complex form was inert toward SKW6.4 cells. The differential dose-dependent cellular binding events could be visualized through the shifting of histograms on flow cytometry, exemplified by 2 mAb concentrations at 0.625 and 10 μg/mL.

Overall, the observations from ELISA and SKW6.4 cells confirm that free UB-221 (or IgE-complexed form) can interact unrestrictedly with the IgE-CD23 complex (or CD23). Free ligelizumab can react with the IgE-CD23 complex only limitedly, while in the IgE-mAb complex form it loses its capability to bind CD23. Omalizumab in either free or complexed form is completely inert toward CD23.

### Molar ratio–dependent formation of UB-221–IgE complex oligomers.

**Figure 3:**
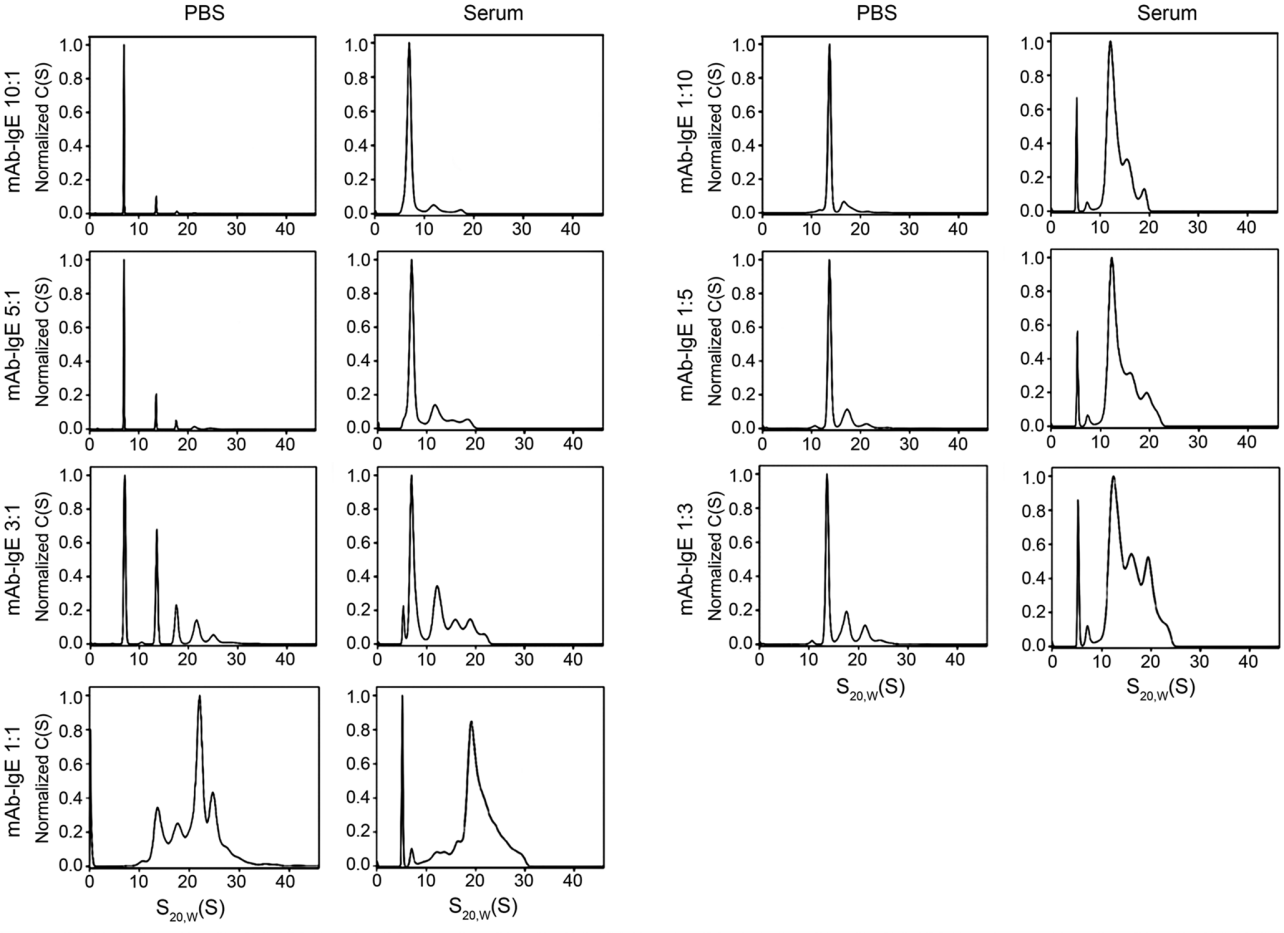
Formation of UB-221–IgE complexes in PBS and human serum. A study of UB-221–IgE complex formation in PBS solution and human serum was conducted using fluorescence detection system–analytical ultracentrifugation (FDS-AUC), in which the Alexa Fluor 488–conjugated UB-221 was used as a marker. The human IgE and UB-221 were mixed in PBS and serum at a dynamic range of molar ratios from 1:1 through 1:10, and the formed complexes were analyzed by FDS-AUC as described in the Methods. The overall results of FDS-AUC suggest that a molar excess of either UB-221 or IgE would result in the formation of smaller complexes, while the largest complexes are formed at an equimolar ratio; the presence of UB-221–IgE complexes in an equimolar ratio at x:y or y:x (e.g., 1:3 or 3:1) would produce a similar complex pattern; and complex profiles in PBS and serum are similar, except that broadening peaks are seen associated with serum samples likely due to higher viscosity and other physicochemical mechanisms. C(S) represents the sedimentation coefficient distribution values with 68% confidence level; S_20,W_(S) represents the apparent sedimentation s-values that were converted to s20,W using density and viscosity of the buffer solutions measured on densitometer and viscometer.

Of note, the UB-221–IgE complex could bind to CD23 (Figure 2, C and D) with different mAb-IgE oligomers. Using Alexa Fluor 488–conjugated UB-221 as a marker and probed with the fluorescence detection system–analytical ultracentrifugation (FDS-AUC) method (42, 43), we explored the species of the UB-221–IgE immune complex that may form in PBS and in human serum at various molar ratios, to mimic the clinically relevant serum mAb concentration range upon UB-221 dose administration. The oligomeric complex patterns were essentially similar between PBS and serum (Figure 3), except for the relatively broadened peaks seen in serum that are likely due to higher viscosity, preferential solvation, and nonspecific association with serum components, as previously reported (43).

In PBS and serum, at a 1:1 molar ratio, the most abundant complex probably corresponds to the 3:3 heterohexamer (UB-221)_3_(IgE)_3_ detected at approximately 21.5 S (Figure 3). A 3-fold excess of IgE over mAb (UB-221/IgE at 1:3) resulted in the formation of smaller complexes, with a major complex shifted to approximately 13 S. A 5-fold and 10-fold excess of IgE (UB-221/IgE at 1:5 and 1:10) resulted in a further decrease in larger complexes with a concomitant increase in the area under the approximately 13 S peak, which likely represents the heterotrimer (UB-221)_1_(IgE)_2_. When mAb was in excess, smaller complexes and a large peak at approximately 7 S corresponding to the monomeric IgE were detected; a major mAb-IgE complex was detected at approximately 13 S and likely represents the heterotrimer (UB-221)_2_(IgE)_1_.

Overall, a molar excess of either UB-221 or IgE would result in the formation of smaller complexes, while the largest complexes are formed at an equimolar ratio. The complex form would thus predominantly exist as the 1:1 heterodimer (UB-221)_1_(IgE)_1_ when the UB-221/IgE molar ratio is 10:1, 50:1, or higher. Pharmacokinetically, a single UB-221 dose would result in a dynamic range of mAb/IgE ratios spanning well over 10:1, with which the 1:1 heterodimer would predominate, in particular under a repeat-dose regimen in which most of IgE the human body produces would be rapidly mopped up by UB-221.

### UB-221 significantly inhibits IgE neosynthesis at both the protein and mRNA level.

We investigated how the binding to CD23 would influence IgE synthesis in PBMCs of healthy donors under costimulation with IL-4 and an anti-CD40 antibody, which are known not only to trigger class switching of IgE-positive B cells but also to induce expression of CD23 (44, 45). Using a previously described PBMC IgE study methodology (46), the de novo IgE protein synthesis is generally quantifiable starting on day 7 and reaches a maximum and plateau at around days 11–14 (Supplemental Figure 5).

The validity of the PBMC IgE method was tested in the UB-221-versus-omalizumab study at concentrations of 1, 3, and 10 μg/mL, showing UB-221’s superiority over omalizumab on days 7 and 11, with IgE reduction of 87%–94% for UB-221 and 7.9%–53.3% for omalizumab (Supplemental Figure 6, A–C, and Supplemental Table 1A). However, the production of IgM (Supplemental Figure 6, D–F) and IgA (Supplemental Figure 6, G–I) were not affected, which also implies that the IgG production was not altered. These observations suggest that the impact on de novo protein synthesis is IgE isotype specific and B cell type specific.

**Figure 4:**
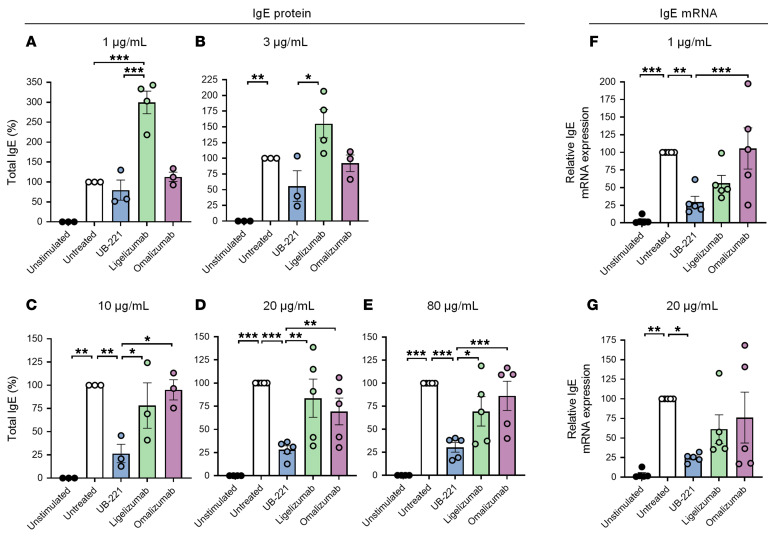
UB-221 induces a pronounced inhibition of IgE protein production and IgE mRNA expression in human PBMCs. In the presence of UB-221, omalizumab, or ligelizumab (UBP lot), human PBMCs from 3 to 5 healthy donors were stimulated with human recombinant IL-4 and an anti–human CD40 antibody to undergo de novo IgE synthesis. (**A**–**E**) The effects on IgE protein production were focused on day 11 by each anti-IgE mAb at doses of (**A**) 1 μg/mL (*n =* 3), (**B**) 3 μg/mL (*n* = 5), (**C**) 10 μg/mL (*n =* 5), (**D**) 20 μg/mL (*n =* 5), and (**E**) 80 μg/mL (*n =* 5). The total IgE in cell culture supernatant samples was quantified by ELISA. (**F** and **G**) In a separate set of donors (*n =* 5), the effects on IgE mRNA expression in lysates of PBMCs stimulated with human recombinant IL-4 and anti–human CD40 antibody in the presence of UB-221, ligelizumab, or omalizumab were studied at (**F**) 1 μg/mL or (**G**) 20 μg/mL. The mRNA expression in cell cultures on day 11 was analyzed by real-time PCR. The percentages of IgE protein/mRNA reduction were calculated based on the total IgE/mRNA levels from the respective untreated cells, which were set as 100%. The percentage reduction values of IgE protein and IgE mRNA are shown in Supplemental Table 1B and Supplemental Table 2, respectively. Data are shown as mean ± SEM. Different treatments were compared relative to the untreated group using 2-way ANOVA with Tukey’s multiple comparison test referenced to untreated controls. *P < 0.05; **P <0.01; ***P < 0.001.

We then defined the effects of the 3 anti-IgE mAbs on the PBMC IgE production at protein and mRNA levels simultaneously in the presence of UB-221, ligelizumab, or omalizumab at a wider concentration range (1, 3, 10, 20, and 80 μg/mL) and focused on day 11, which marked the total IgE accumulated for a time period of 11 days. The results confirm that UB-221 is superior to both omalizumab and ligelizumab in suppression of IgE protein production, relative to the PBMCs “untreated” with mAbs (Figure 4, A–E). UB-221 dose dependently reduced the total IgE level by up to 69%–74% at dose levels of 10 μg/mL or higher, versus a lower 16%–31% for ligelizumab and 5.0%–31% reduction for omalizumab (Supplemental Table 1B).

At the lower 1 and 3 μg/mL dose levels, none of the mAbs showed a statistically significant lowering effect on IgE synthesis, although UB-221 exhibited a trend of 20% to 50% reduction. Ligelizumab appeared to induce a greater IgE reduction than omalizumab, although none of the dose settings showed statistically significant differences (Figure 4, A–E, and Supplemental Table 1B). Exceptionally, an increase in IgE production was notable with ligelizumab at the 1 and 3 μg/mL levels. Little IgE production could be observed in PBMCs “unstimulated” with IL-4 and an anti-CD40 antibody that can induce CD23 expression (44, 45), suggesting that the de novo IgE synthesis driven by an anti-IgE mAb involves CD23.

The isotype-specific suppression of IgE synthesis was observed at the mRNA level as well in PBMCs from a different set of 5 individuals. The effect of mAbs on IgE mRNA expression at doses of 1 and 20 μg/mL was investigated, with a focus on day 11. Unlike the accumulated total IgE protein produced over time, this represents the level of mRNA at a specific single time point. The result reveals that, in parallel with the reduction in fresh IgE protein production, UB-221 significantly suppressed IgE mRNA expression by 70%–75% (Figure 4, F and G), which is far greater than the inhibition by ligelizumab and omalizumab (Supplemental Table 2).

### Apoptosis of membrane-bound-IgE B cells induced by the binding with anti-IgE mAbs.

**Figure 5:**
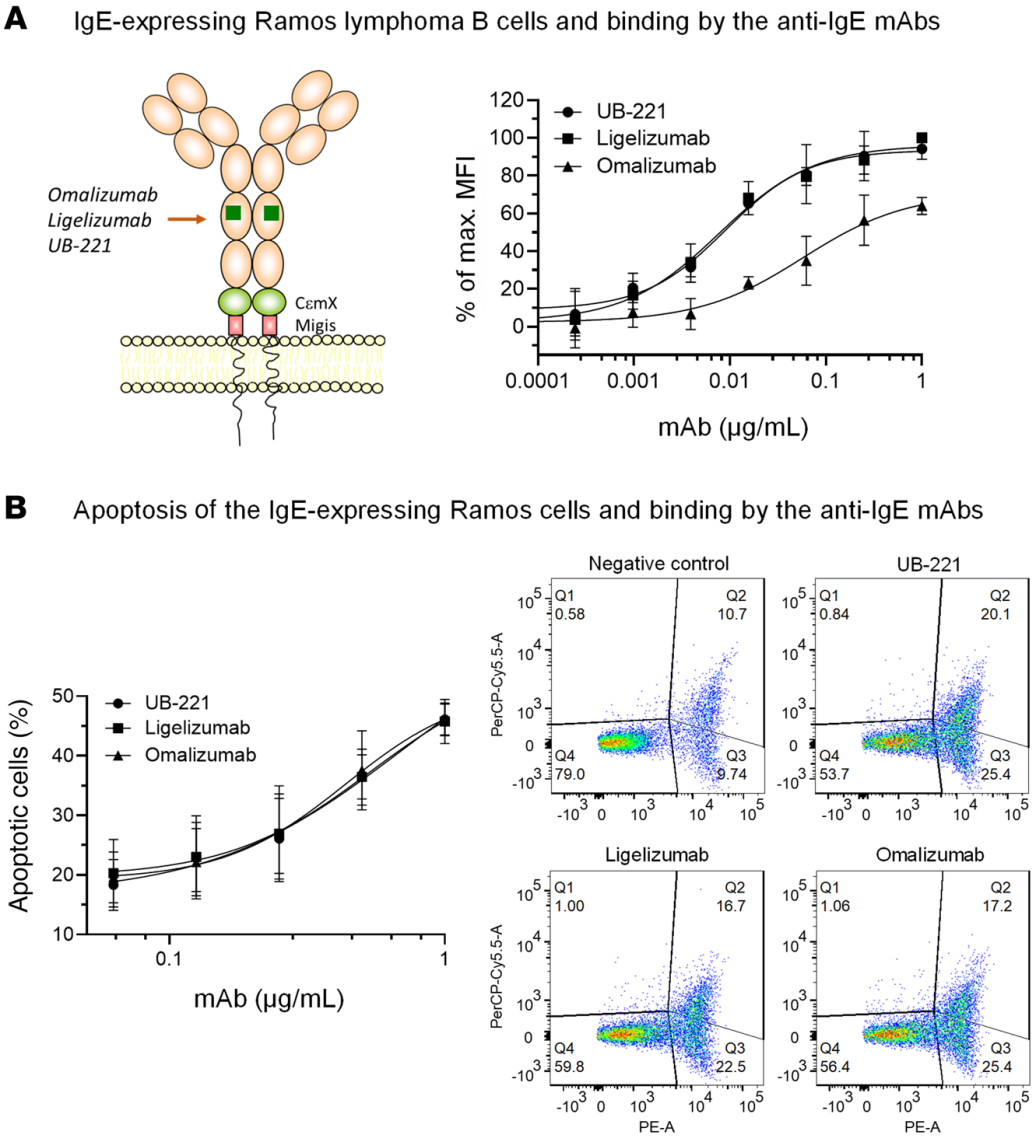
Binding to IgE-bearing Ramos lymphoma B cells and induction of apoptosis by anti-IgE mAbs. (**A**) Ramos lymphoma B cells were transfected to express the Fc portion of a long form of mIgE on the membrane (mIgE.FcL) containing the CεmX domain and Migis stalk, on which the epitopes at the Cε3 and CεmX domains can be targeted by the mAbs as indicated. UB-221, ligelizumab (UBP lot), and omalizumab can bind to the mIgE.FcL-expressing Ramos cells with different binding activities showing that, estimated by EC_50_ values (ng/mL, mean ± SD, *n =* 3), UB-221 (7.9 ± 4.5) and ligelizumab (8.8 ± 3.6) bind to the cells equally strongly with superimposable binding curves, nearly 7-fold greater than omalizumab (55.3 ± 24.6). However, (**B**) the apoptotic effects on the cells induced by the 3 mAbs did not show an apparent difference as revealed by the superimposed cell-death curves. These are also visualized with the cells stained with annexin V–PE and 7-AAD in a representative setting treated with the mAbs at 1 μg/mL, where the same magnitude of shift in cell populations shown in dot plots is notable. The untreated cells were used as a negative control.

The IgE synthesis reduction events (Figure 4, A–E) may be partially attributed to the apoptotic effect resulting from the mAb binding to the IgE class–switched lymphoblasts or membrane-bound-IgE (mIgE) memory B cells within the PBMC population. This was simulated with Ramos B cells expressing a long form of mIgE (46), to which UB-221 and ligelizumab bound equally strongly, with a 7-fold higher affinity than omalizumab (Figure 5A) as evidenced by mean EC_50_ values at 7.9, 8.8, and 55.3 ng/mL for UB-221, ligelizumab, and omalizumab, respectively.

However, all 3 mAbs cause the same degree of apoptosis (Figure 5B), as shown by the similar apoptotic cell death rates. Nonetheless, neither did IgE-binding activity correlate with apoptotic outcome nor did apoptosis align with the ranking order of IgE synthesis inhibition (Figure 4, A–E). These results suggest that apoptosis, if it did occur in the PBMC IgE study system, would be an insignificant factor.

### SPR-based IgE-binding affinity and ELISA-based IgE-neutralizing activity of anti-IgE mAbs in buffer solution.

**Figure 6:**
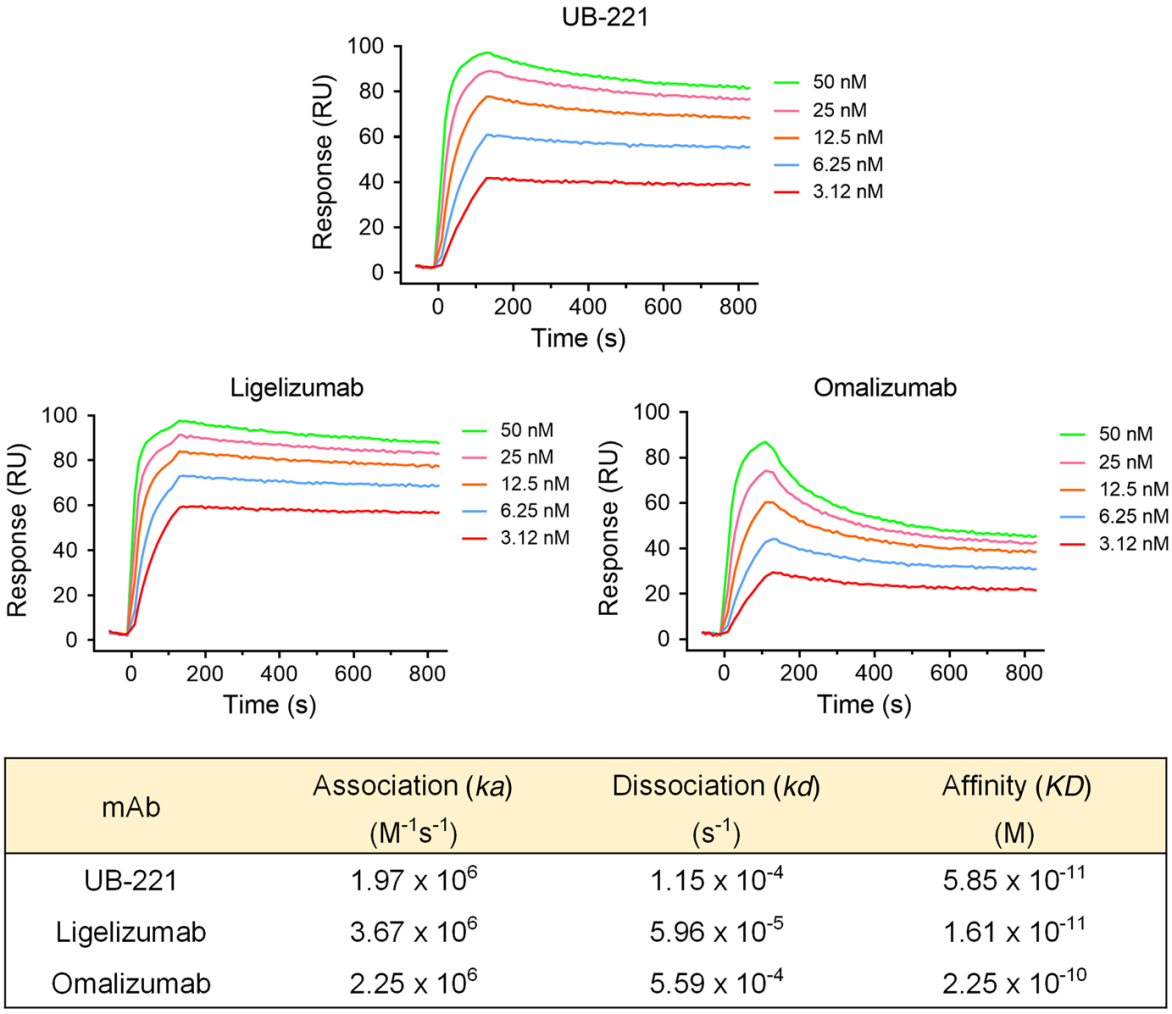
The IgE-binding affinity of UB-221 and ligelizumab on SPR. Kinetic analyses on SPR of IgE binding to the anti-IgE mAbs UB-221, ligelizumab (Creative Biolabs, TAB755), and omalizumab were performed on SPR. IgG Fc antibodies were first covalently coupled to the surface sensor chip, on which the diluted anti-IgE antibodies were captured. The Expi293-expressed human full-length IgE at 0.312–50 nM was injected and the dissociation was measured under constant buffer flow. The resulting equilibrium dissociation rate constant (*K_D_*, binding affinity), association rate constant (*k_a_*), and dissociation rate constant (*k_d_*) are presented in the table.

In addition to the cellular IgE targeting on Ramos B cells where UB-221 and ligelizumab were observed to bind with an equal affinity (expressed as EC_50_) and omalizumab with lower affinity (Figure 5A), the 3 mAbs were compared in surface plasmon resonance (SPR) analysis where they were immobilized by an anti–human Fc covalently coupled on the sensor chip, over which human IgE flowed (Figure 6). Compared with omalizumab, UB-221 showed a binding affinity (*K_D_*, 5.9 × 10^–11^ M) that was approximately 4-fold stronger than that of omalizumab (*K_D_*, 2.3 × 10^–10^ M), and a dissociation rate (*k_d_*, 1.2 × 10^–4^ s^–1^) that was approximately 5-fold lower than that of omalizumab (*k_d_*, 5.6 × 10^–4^ s^–1^).

Ligelizumab exhibits the strongest affinity (*K_D_*, 1.6 × 10^–11^ M), which was approximately 4-fold and approximately 14-fold greater than that of UB-221 and omalizumab, attributable to a dissociation rate (*k_d_*, 6.0 × 10^–5^ s^–1^) that was approximately 5-fold and 10-fold lower than that of UB-221 and omalizumab, respectively. No significant differences were apparent in the association rates (*k_a_*, 2.0 × 10^6^ to 3.7 × 10^6^ M^–1^s^–1^) among the 3 antibodies.

The binding affinity (*K_D_*, 1.6 × 10^–11^ M) of the ligelizumab used in the present SPR analysis (Figure 6) is very similar to the recently reported value for the “original” ligelizumab (*K_D_*, 1.8 × 10^–11^ M) (35). The latter was obtained in a different experimental setting with IgE immobilized through FcεRI-Fcγ covalently attached to the sensor chip, over which the mAbs flowed. Their other binding attributes, such as dissociation rates (*k_d_*, 3.3 × 10^–5^ vs. 6.0 × 10^–5^ s^–1^) and association rates (*k_a_*, 1.8 × 10^6^ vs. 3.7 × 10^6^ M^–1^s^–1^) (35), do not reveal significant differences from the counterparts obtained in the present study. The ligelizumab used in the present study could legitimately represent the original ligelizumab as far as the IgE binding or IgE neutralization is concerned.

Unlike the case of UB-221 versus omalizumab where their differential IgE binding affinities on SPR (Figure 6) translate well into their respective functional IgE-neutralizing potency (Figure 1, B–D), an antigenic 5-fold SPR “binding” superiority of ligelizumab over UB-221 does not amount to a stronger functional “neutralization.” In fact, UB-221 and ligelizumab in PBS (from 2 United BioPharma [UBP] in-house lots and from Creative Biolabs) were equipotent in competitive inhibition of IgE binding to FcεRI on ELISA, as evidenced by their superimposable IgE-neutralizing curves (Supplemental Figure 7). A similar neutralizing equipotency has also been confirmed in suppressing a high level of IgE in sera from patients with AD, as described below.

### UB-221 and ligelizumab neutralize high IgE in sera of patients with AD with equal strength.

**Figure 7:**
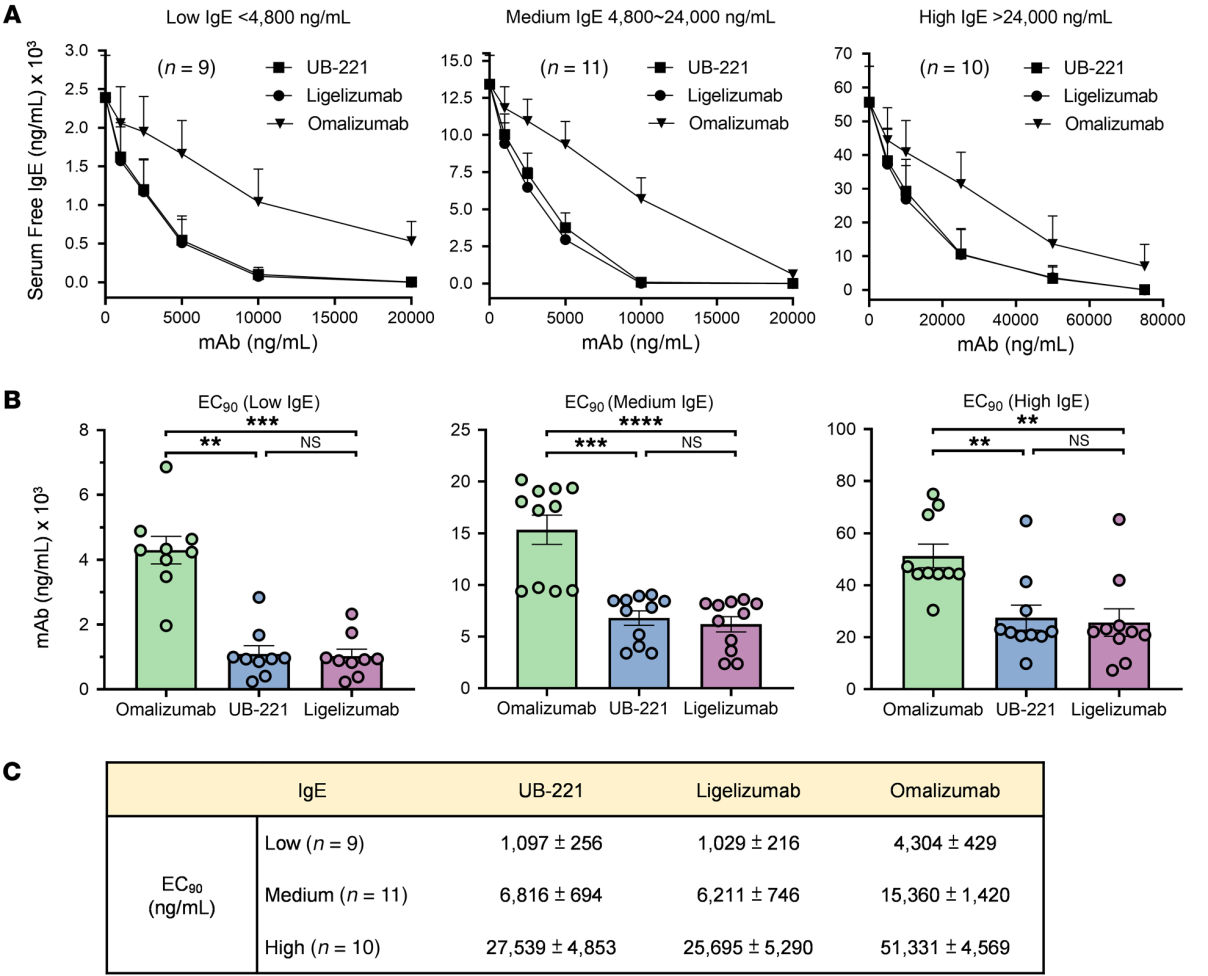
Ex vivo reduction of high-level IgE in sera of patients with atopic dermatitis. The potency in reducing high IgE in sera from 30 patients with atopic dermatitis was compared for UB-221, ligelizumab (Creative Biolabs, TAB755), and omalizumab, based on a competitive inhibition of IgE binding to FcεRI immobilized on ELISA solid phase. The serum samples were incubated for 1 hour at room temperature with 3 increasing concentrations of anti-IgE mAbs. The remaining free IgE in sera was quantified as described in the Methods. (**A**) The serum samples collected were grouped into 3 IgE ranges of low (<4,800 ng/mL, *n =* 9), medium (4,800–24,000 ng/mL, *n =* 11), and high IgE (>24,000 ng/mL, *n =* 10). (**B**) The mAb drug concentrations to achieve a reduction in serum IgE of 90% of the basal IgE levels are illustrated and (**C**) the EC_90_ values are presented. All data are shown as mean ± SEM for the low-, medium-, and high-IgE groups. The comparisons were estimated by the Kruskal-Wallis test method. **P < 0.01; ***P < 0.001; ****P < 0.0001. NS, not significant.

As IgE levels are often very high in patients with AD, we investigated the efficacy of the mAbs in lowering the high IgE in sera of patients with AD. Of 30 patients with AD, their basal serum IgE levels were grouped into 3 different ranges: low (<4,800 ng/mL), medium (4,800–24,000 ng/mL), and high (>24,000 ng/mL) (Figure 7). The serum samples were incubated with UB-221, ligelizumab, or omalizumab at 3 increasing concentration ranges. The results indicate that UB-221 and ligelizumab performed equipotently in lowering serum IgE levels across all baseline-IgE-range groups, as shown by the superimposable neutralization curves (Figure 7A), while omalizumab is apparently a weaker performer.

For the low-baseline-IgE group of patients with AD, to neutralize 90% of IgE (EC_90_) (Figure 7B) may require 1.0 μg/mL UB-221 or ligelizumab, while a more than 4-fold greater concentration, 4.3 μg/mL, would be needed for omalizumab (Figure 7C). The fold differences narrow to 2.5- and approximately 2.0-fold in the medium- and high-baseline-IgE groups, respectively. For example, with the high-baseline-IgE range, to neutralize 90% of IgE would require more than 25 μg/mL UB-221 or ligelizumab, while that for omalizumab could be more than 50 μg/mL.

The overall ex vivo data exemplified with sera of patients with AD implies that, given a potent IgE neutralizer for treatment of IgE-sensitive allergic diseases, it remains a high-hurdle task to bring down the high serum-free IgE (and so allergen-specific IgE) close to a baseline, single-digit level if the steady, high IgE supply from neosynthesis could not be kept under effective control with measures in addition to simple neutralization of IgE.

### UB-221 at a single dose induces rapid, pronounced reduction in serum free IgE in cynomolgus macaques and hIGHE-knockin mice.

**Figure 8:**
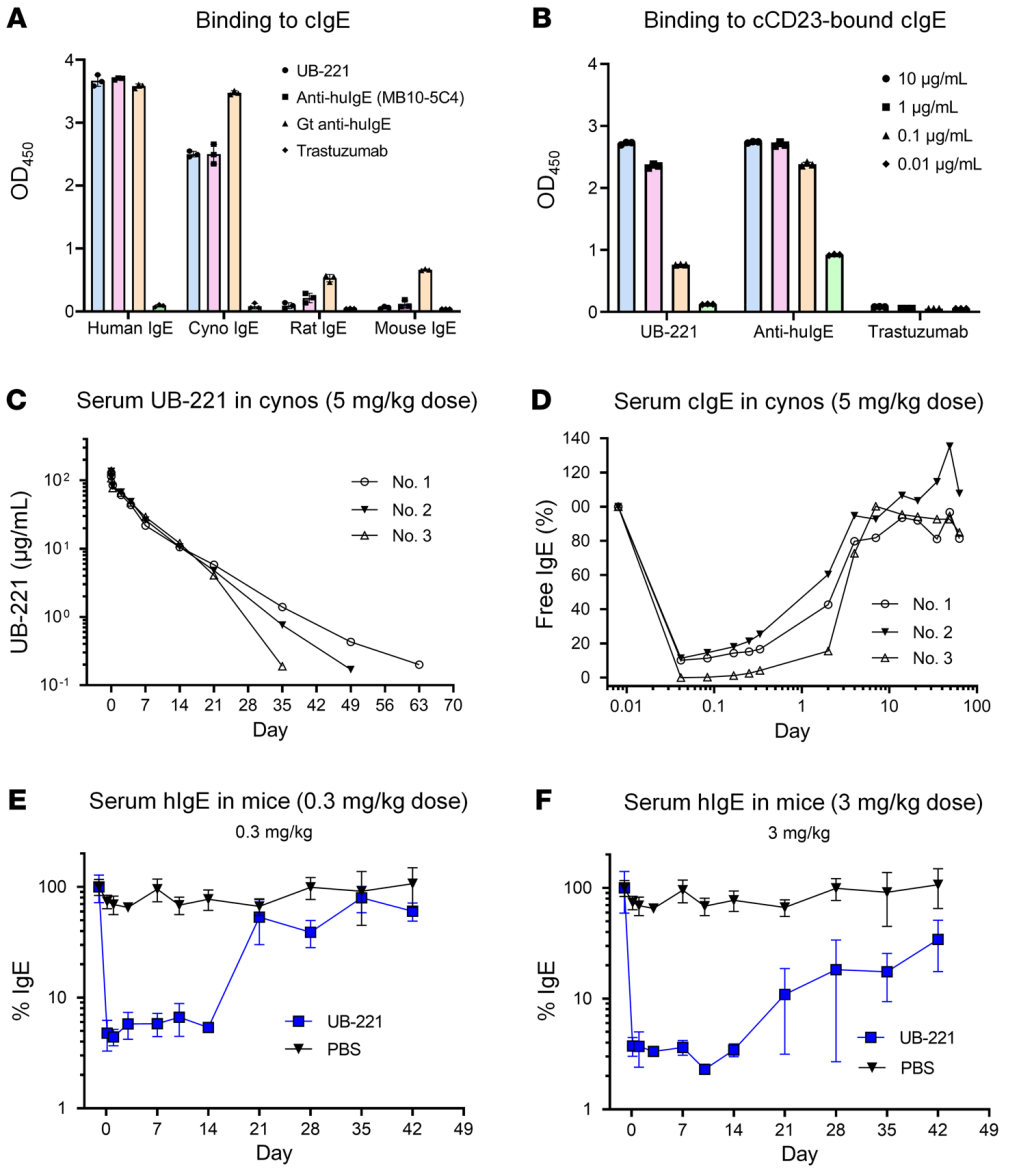
A rapid and pronounced serum free-IgE reduction in cynomolgus macaques and hIGHE-knockin mice after a single i.v. infusion dose of UB-221. UB-221 can (**A**) bind to cynomolgus macaque IgE (cIgE), but not rat or mouse IgE, and can also (**B**) bind to the cCD23-bound cIgE. (**C**) In cynomolgus macaques (cynos, *n =* 3) receiving a single i.v. dose at 5.0 mg/kg, UB-221 decayed over time, with a mean half-life of approximately 6.3 days, in which (**D**) UB-221 was able to induce a rapid reduction in serum free cIgE by 90%–100% in the treated macaques. The basal cIgE levels were at the range of 399 to 434 ng/mL for the 3 macaques. In hIGHE-knockin mice (*n =* 6 per dose group), a single i.p. dose of UB-221 (**E**) at 0.3 mg/kg or (**F**) at 3.0 mg/kg can induce a rapid, greater than 95% reduction in serum free chimeric IgE (mean ± SEM). The basal chimeric IgE levels in the hIGHE-knockin mice were at the range of 416 to 1,365 ng/mL for the 0.3 mg/kg dose group, and of 734 to 3,362 ng/mL for the 3.0 mg/kg dose group.

UB-221 can bind to cynomolgus macaque IgE (cIgE) (Figure 8A) and engage cynomolgus macaque CD23–bound (cCD23-bound) cIgE (Figure 8B), demonstrating that the cynomolgus macaques can serve as an appropriate pharmacology and toxicology animal model. In cynomolgus macaques (*n =* 3) receiving a single intravascular dose of UB-221 at 5.0 mg/kg, the serum antibody concentration declined, with a mean elimination half-life of 6.3 days (Figure 8C), in which UB-221 could induce a rapid, pronounced reduction in serum free cIgE by 90% to 100% (Figure 8D).

Potent in vivo inhibition of serum IgE was evidenced as well in hIGHE-knockin mice, in whose genome the Cγ1 constant region is replaced by the human Cε constant region (47). The mice yield IgG_1_ class-switching B cells that can produce chimeric IgE. In these mice (*n =* 6) receiving a single i.p. dose (Figure 8E) at 0.3 mg/kg or at 3.0 mg/kg (Figure 8F), UB-221 can rapidly reduce the high serum free-chimeric-IgE level by greater than 90%.

### A single UB-221 dose administered to patients with CSU safely improved disease symptoms along with rapid reduction in serum free-IgE level.

A phase I, open-label, dose-escalation trial to evaluate the safety, tolerability, pharmacokinetics, and pharmacodynamics of a single i.v. infusion of UB-221 as an add-on therapy was conducted in patients with CSU under first-line H1-antihistamine treatment (ClinicalTrials.gov NCT03632291). The study participants in 5 dose cohorts (0.2, 0.6, 2.0, 6.0, or 10 mg/kg; *n =* 3/cohort, total 15 study participants) (Supplemental Table 3) were monitored for 14 weeks after dosing. UB-221 was safe and well tolerated up to the highest investigated dose at 10 mg/kg; no study participant experienced dose-limiting toxicity during the entire study period; no serious adverse event, death, serious and unexpected suspected adverse reaction, or infusion reactions were observed (Supplemental Table 4).

**Figure 9:**
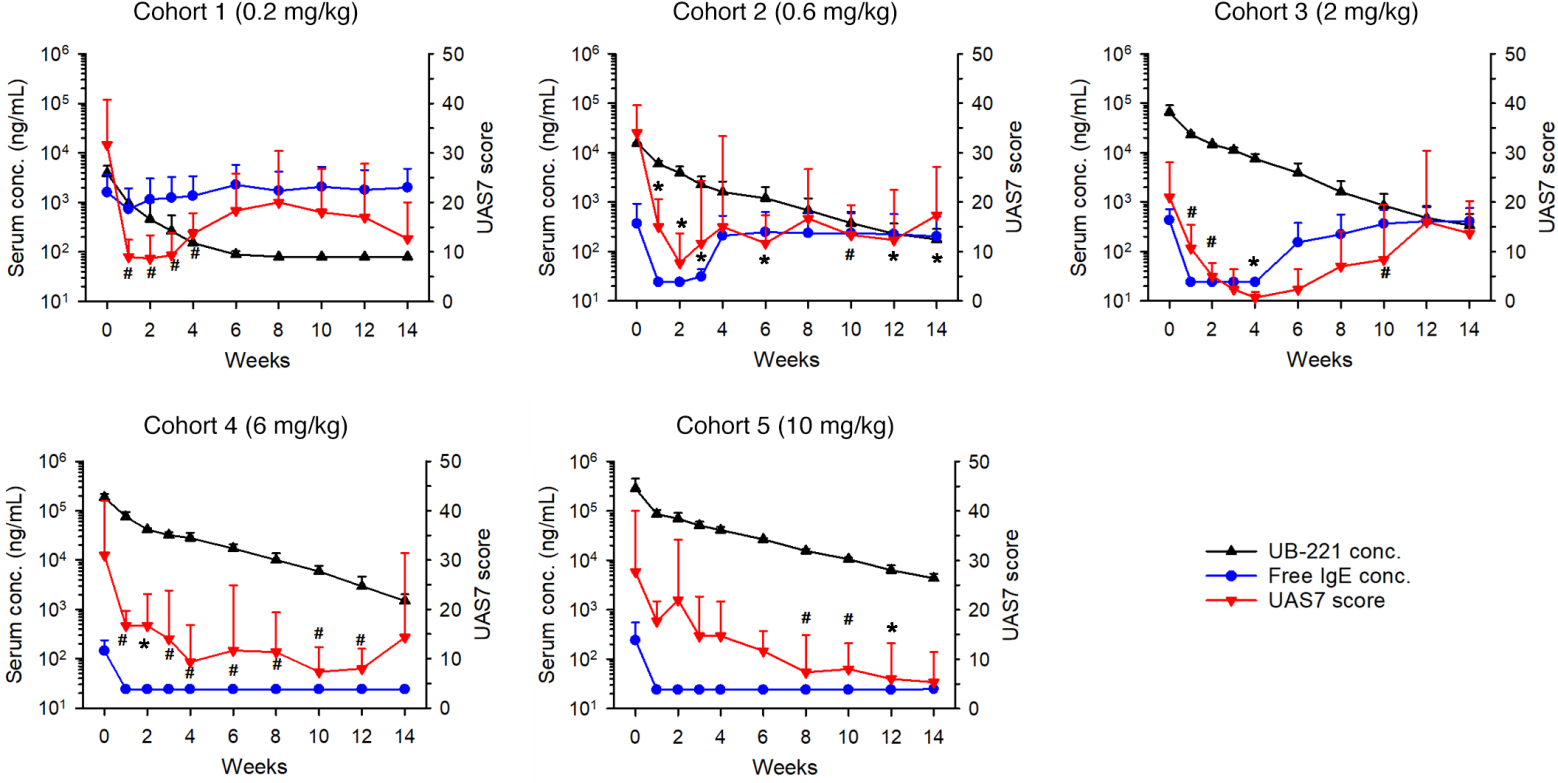
Concurrent serum UB-221 concentration, serum free-IgE level, and UAS7 disease score in patients with CSU after a single i.v. dose of UB-221 in phase I trial. Shown in parallel are the averaged (mean ± SD) serum concentrations (ng/mL) for both UB-221 (black line) and free IgE (blue line), and CSU symptom relief (reduction) expressed as UAS7 scores (red line) in study participants of 5 dose cohorts (0.2 to 10 mg/kg; *n =* 3 per cohort) over 14 weeks of the phase I single i.v. UB-221 dose trial. The postdose CSU symptom relief score UAS7 is a combined efficacy marker of hives severity score over 7 days (HSS7) and itch severity score over 7 days (ISS7). The half-life of UB-221 was estimated to be in the range of 16 to 22 days at doses of 0.6 to 10 mg/kg (Supplemental Table 3). The UAS7 changes (red curves) relative to the baseline were estimated and compared by the paired *t* test for significant differences. Due to the small sample size in each dose cohort that had only 3 participants, P < 0.1 was also assigned to indicate the trend of positive efficacy measured by the decrease in UAS7. ^#^*P <* 0.1, *P < 0.05.

A single UB-221 dose, which decreased dose dependently in circulation with a half-life estimated to be 16 to 22 days at doses of 0.6 to 10 mg/kg (Supplemental Table 5), induced a dramatic reduction in serum free-IgE level in parallel with a rapid, dose-dependent decrease in weekly UAS7, i.e., disease symptom relief (Figure 9). The mean UAS7 scores (±SD) at week 0 baseline (and at the week lowest after disease symptom relief) were 31.7 ± 8.7, 34.0 ± 7.7, 21.0 ± 0.7, 31.0 ± 7.0, and 27.7 ± 5.7 for respective dose cohorts from 0.2 through 10 mg/kg, respectively. At higher UB-221 concentrations in the 6 and 10 mg/kg cohorts, the free-IgE levels were suppressed fully and the reduction in UAS7 disease scores persisted for a longer period of time. The IgE-UAS7 correlation suggests that a single dose of more than 2.0 mg/kg could potentially allow UB-221 to be administered every 3 to 6 months and achieve a complete response (UAS7 = 0) or well-controlled stage (UAS7 ≤ 6) in the treatment of CSU. The results suggest that, in patients with CSU, the level of serum free IgE plays a critical role.

From the perspective of individual study participants, in all 13 participants with moderate to severe CSU (UAS7 score ≥ 16) at baseline, a rapid reduction in UAS7 occurred in the first week (Supplemental Figure 8A); relief of symptoms to a well-controlled/disease-free stage (UAS7 ≤ 6) appeared to be dose dependent and achieved in 10 out of the total 15 participants. In particular, all 3 participants in the 2 mg/kg cohort experienced a sustained disease-free stage, i.e., complete response (UAS7 = 0) for 2 to 4 weeks after dosing, and a sustained hive-free stage (weekly hives severity score, HSS7 = 0) for 3 to 12 consecutive weeks (data not shown). The UB-221–mediated dose-dependent UAS7 profiles largely correlate with the durable suppression of serum free IgE and slow return to their respective baseline IgE level (Supplemental Figure 8B), which parallels as well with the reduction in FcεRI on basophils (Supplemental Figure 8C).

## Discussion

The finding that FcεRI and CD23 function mutually exclusively due to reciprocal allosteric conflict (9, 16–18) supports the contention that the inflammatory FcεRI preferentially binds free IgE to drive allergic hypersensitivity, while the noninflammatory CD23 preferentially engages allergen-IgE complexes to downregulate IgE through mechanisms of IgE clearance and IgE synthesis reduction via negative regulation of BCR signaling (28, 29).

While the consequences of interaction between IgE-specific antigens (allergens or autoantibodies) and IgE-FcεRI have been well known, precisely how a free-IgE–targeting mAb would engage CD23-bound IgE and how an IgE-complexed mAb would bind to CD23 in an unrestricted fashion have been relatively unexplored. UB-221 represents such an unprecedented, unique anti-IgE mAb of a newer class that presents 4 major preclinical findings. In addition, UB-221 in the phase I single-dose clinical trial with CSU patients has shown durable disease symptom relief along with rapid reduction in serum free IgE.

First, UB-221 is superior to omalizumab over a host of IgE-neutralization studies. Without causing activation of human basophils (Figure 1A), UB-221 can inhibit the IgE-OVA–induced degranulation in RBL SX-38 basophils (Figure 1B), potently reduce high IgE in sera of patients with AD ex vivo (Figure 7), and can induce rapid, pronounced reduction in serum IgE in hIGHE-knockin mice (Figure 8, E and F).

One notable superiority of UB-221 is in alignment with the greater direct IgE-binding affinity (*K_D_*) measured by SPR (Figure 6), the higher binding (EC_50_) to the IgE-bearing Ramos B cells (Figure 5A), and with the competitive edge in inhibition of IgE binding to FcεRI observed on FcεRI-immobilized ELISA (Figure 1C) and FcεRI^+^ RBL SX-38 basophils (Figure 1D).

Second, UB-221 and ligelizumab perform with equal strength in both cellular IgE binding and IgE neutralization. They bind mIgE^+^ B cells (Figure 5A) and neutralize soluble IgE in buffer solution (Supplemental Figure 7) and high IgE in sera of patients with AD (Figure 7) equally strongly, despite differing in IgE binding by SPR (Figure 6), which shows a 4-fold lower affinity for UB-221 observed under a covalent sensor-chip-coupling setting different from that reported earlier for ligelizumab (35).

That the binding affinity by SPR is not consistent with functional activity for the case of UB-221 versus ligelizumab may be understood by the fact that the equilibrium affinity on SPR solid phase, binding to cellular membranes, and competitive neutralization in liquid phase represent 3 differential analytical modes. This inconsistency may be better yet explained via differential spatial conformations upon IgE interactions of the 3 anti-IgE mAbs and FcεRI.

**Figure 10:**
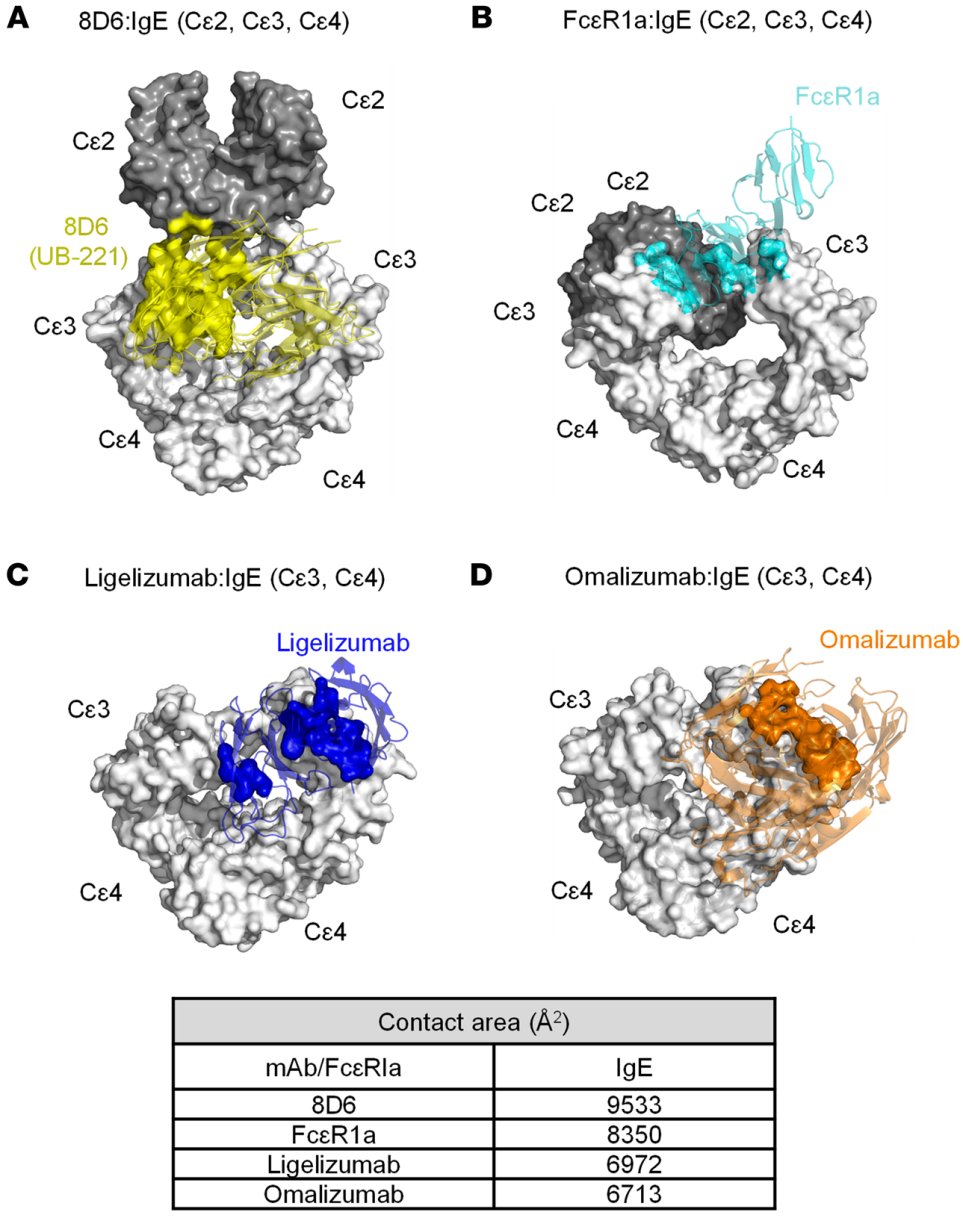
Superposed 3D structures of the mAb-IgE complex and contact interfaces of IgE bound with the 3 mAbs and FcεR1α. The 3D illustrations of 3 mAb-IgE and FcεR1α-IgE complex structures were generated using the Protein Data Bank accession numbers 6EYO (8D6:IgE), 6UQR (ligelizumab:IgE), 5HYS (omalizumab:IgE), and 2Y7Q (FcεR1α:IgE). 8D6 (yellow), ligelizumab (blue), omalizumab (brown), and FcεR1α (cyan) are shown in ribbon format. The structures were generated using the PyMOL program. IgE molecule is shown in surface format with Cε2 (dark gray) and Cε3-Cε4 (gray). (**A**) The Fab of 8D6 contacts both the Cε2 domain and Cε2-Cε3 linker of the IgE molecule (yellow), which exhibits a fully extended conformation. (**B**) FcεR1α binds the Cε3 domain of IgE, which adopts a bent conformation. (**C**) Ligelizumab (blue) and (**D**) omalizumab (brown) bind an IgE molecule with similar contact areas. Interfacial areas were calculated with AREAIMOL in the CCP4 program suite. The contact areas were calculated on the van der Waals surface of an atom within 3.8 Å. 8D6 interfaces with IgE with the greatest contact area (9,533 Å^2^), followed by FcεR1α (8,350 Å^2^), ligelizumab (6,972 Å^2^), and omalizumab (6,713 Å^2^).

Ligelizumab and omalizumab share a large swath of binding site overlap on the Cε3 domain, with similar interfacial contact areas and near the Cε2 domain (Figure 10, C and D), and as such, they both inhibit IgE binding to FcεRI (Figure 10B) due to allosteric conflict, as reported previously (23, 35, 48). Both antibodies would therefore exhibit a proportional antigenic-functional relationship in inhibition of IgE binding to FcεRI (neutralization) according to their scale of differential binding affinity.

On the other hand, 8D6 (the parent murine mAb of UB-221) binds to IgE through a mixed protein-carbohydrate epitope (36), which involves contacts with the Cε2 and Cε3 domains and is associated with further flexibility and a novel extended conformation (Figure 10A). Thus, steric conflicts exist between 8D6 and FcεRI, and the (Cε2)_2_ domain pair and FcεRI, and the inhibition of IgE binding to FcεRI by 8D6 (and so by UB-221) involves both allosteric and orthosteric hindrances, as previously reported (36). These observations lay the foundation for eliciting a high level of IgE-neutralizing activity in buffer solution (Supplemental Figure 7) and in sera (Figure 7) with a potency equal to that of ligelizumab.

Third, UB-221 in free form (or IgE-complexed form) can interact unrestrictedly with IgE-CD23 complex (or CD23) (Figure 2), a unique feature that to the best of our knowledge has not previously been observed for any anti-IgE mAb. The interactions with CD23 could be visually verified through the superposed mAb-IgE-CD23 complex structures (Supplemental Figure 9). CD23 binds to the epitopes at the juncture of the Cε3 and Cε4 domains of IgE. The 8D6 mAb (the murine parent precursor of UB-221) can bind to a site on IgE that is distant from where IgE contacts CD23 (Supplemental Figure 9A), with the surface representation of 8D6 binding showing no steric conflict with the IgE-CD23 interaction zone. This supports the idea that 8D6 (and so UB-221) can bind freely to CD23-bound IgE (Figures 2A and 2B) and that UB-221 in IgE immune complexes can interact unrestrictedly with CD23 (Figure 2, C and D).

Ligelizumab binds to the site on IgE that partially overlaps the sites where IgE and CD23 interact (Supplemental Figure 9B), and the surface representation of the ligelizumab binding shows a minor steric conflict with the IgE-CD23 interaction zone. As for omalizumab, it binds to sites on IgE that substantially overlap the sites where IgE and CD23 interact (Supplemental Figure 9C). The atomic volumes (Å^3^) of the overlapping sites have been estimated to be 100-fold different, approximately 28 Å^3^ for ligelizumab versus 2773 Å^3^ for omalizumab (35). These results support the notion that, in free form (Figure 2, A and B) or IgE-complexed form (Figure 2, C and D), ligelizumab presents only minor binding to the CD23-bound IgE on ELISA and the CD23^+^ SKW6.4 cells, and that omalizumab stays totally inert toward CD23.

The CD23-binding observations in ELISA and SKW6.4 cells (Figure 2) and the mAb-IgE-CD23 complex (Supplemental Figure 9) also confirm earlier reports that omalizumab completely “inhibits IgE binding” to CD23 as it blocks the IgE binding to CD23 orthosterically (23, 27), while ligelizumab does so less potently (27, 35, 48). Furthermore, the unrestricted interaction of UB-221 with CD23 is of considerable interest regarding CD23-mediated IgE synthesis in B cells, which any form of the multiple oligomeric mAb-IgE complex species (Figure 3) would be allowed to stack onto and cross-link the CD23 molecule to influence IgE synthesis.

Fourth, UB-221 downregulates the IgE-isotype-specific neosynthesis at both the protein and mRNA level and to a far greater degree than omalizumab and ligelizumab (Figures 4). The superiority of UB-221 could mainly result from modulating the CD23-mediated pathway, and this is consistent with the long-proposed function of CD23 in negative-feedback regulation of IgE synthesis on B cells, caused by cross-linking with natural IgE, anti-CD23 antibody, or IgE-antigen immune complexes (14, 39–41, 49–52). This mimics the effect of aggregates with CD23 on B cells and that by cross-linking CD23 to lead to the reduction in IgE production (49, 50, 53), supporting the idea that the greater the interaction with CD23, the higher the suppression of neo-IgE synthesis. The apoptotic effect against mIgE^+^ Ramos B cells, which was equal for all 3 mAbs (Figure 5), is unlikely to be a significant contributing element to the reduction in IgE synthesis.

The role of CD23 as a regulator of allergic diseases has been overlooked (9) in the contexts of IgE synthesis regulation and multiple other immunological functions (8). Mice deficient in CD23 or carrying mutated CD23 variants show a phenotype of IgE hypersensitivity (41, 54, 55), while reduced serum IgE level and strongly suppressed IgE responses are seen in CD23-overexpressing transgenic mice (56, 57). Further, the metalloprotease ADAM10 can cleave the membrane-bound CD23 and release it as a soluble CD23 (58), which can cross-link mIgE and membrane-bound CD21 to result in enhanced IgE production (59). Thus, theoretically, a stable presence of trimeric CD23 maintained with IgE engaged on B cells would be a desirable natural means for initiating an IgE inhibitory signal to decrease IgE synthesis (9), a process that may be facilitated by UB-221’s avid engagement with CD23.

Given the reported potential to dislodge the bound IgE from FcԑRI (35, 60) when present at a very high concentration, omalizumab works mainly by binding to neutralize IgE and trap it to form mAb-IgE immune complexes for clearance (61). However, omalizumab requires a high-dose regimen to achieve a clinical benefit, a limitation of therapeutic efficacy likely due the fact that omalizumab, in either a free or IgE-complex form, rejects IgE binding to CD23 (27, 35, 48), and as such omalizumab is not involved in the CD23-mediated IgE downregulation mechanism (Figures 2 and 4).

On the other hand, ligelizumab has been previously reported to have advantages over omalizumab, with higher IgE-binding affinity and thus greater IgE-neutralizing activity, which would have yielded a longer and greater IgE suppression in the phase I trial (33) and a better efficacy in the treatment of CSU in the phase IIb trial (34). Despite a long suppression of serum free IgE observed in the single-dose phase I study, ligelizumab shows a much more rapid rebound of serum free IgE to the baseline level as compared with omalizumab (33). Of note, another IgE neutralizer, MEDI4212 mAb, has an IgE-binding affinity that is more than 100-fold stronger than that of omalizumab, as measured by SPR (62); however, in individuals with IgE-associated allergic symptoms, the duration of serum free-IgE suppression is shorter and the rebound of free IgE to baseline is faster than that of omalizumab (63). Development of MEDI4212, which has a short serum elimination half-life of 4–8 days, has been discontinued.

Although ligelizumab has been previously reported to exert a greater reduction in IgE synthesis than omalizumab (35), the IgE-synthesis reduction is of minor significance. A similar minor level of reduction in IgE production is also seen in the present report with ligelizumab (Figure 4). Both omalizumab and ligelizumab would have been operating in the clinic mainly as IgE neutralizers.

The fact that ligelizumab showed anti-CSU greater efficacy compared with omalizumab in the phase IIb (34) but not in the phase III trial (https://www.hcplive.com/view/phase-3-ligelizumab-not-superior-omalizumab) suggests that, as an IgE neutralizer, a sole factor of greater than 10-fold stronger IgE-binding affinity may not necessarily confer a better clinical outcome. In fact, omalizumab has already been operating as a striking high-affinity IgE binder, with a *K_D_* of 2.3 × 10^–10^ M (Figure 6). Although ligelizumab binds IgE with an approximately 4-fold higher affinity than that of UB-221 on SPR (Figure 6), both mAbs bind mIgE on B cells (Figure 5A), and neutralize free IgE on ELISA (Supplemental Figure 7) and serum free IgE in sera of patients with AD (Figure 7), with equal strength. A higher IgE-binding affinity measured on SPR would be just one facet of multiple functions for an antibody like UB-221 that is distinct from omalizumab and ligelizumab with regard to unrestricted interactions between free UB-221 and IgE-CD23 or between UB-221–IgE and CD23 (Figure 2). UB-221 can form moderately sized anti–IgE-IgE complexes that can bind to CD23, which may modulate CD23^+^ B cells as antigen-IgE complexes do (28, 29). The additional properties of UB-221 may mediate different clinical outcomes.

The more UB-221 binds to CD23-occupied IgE, and the more UB-221–IgE complexes bind to CD23, the less IgE would be available for binding to FcεRI on mast cells and basophils, and as such CD23 serves as well to antagonize the allergic, inflammatory path. Further, CD23 could exert a specific role in quickly clearing IgE immune complexes (28), which include allergen-IgE and anti-IgE autoantibody–IgE complexes, suggesting that the more stable a complex, the more the complex binds to CD23, and the faster and greater the CD23-dependent clearance will be. This supports the contention that it could be of benefit to target IgE toward the noninflammatory CD23 pathway instead of blocking this interaction (29). The CD23-mediated clearance path may thus favor UB-221 over ligelizumab and omalizumab.

Although it seems counterintuitive and paradoxical, strong interaction with IgE-CD23 may be associated with a concern for allergic lung inflammation. However, there have been conflicting reports, as such an adverse effect may (64, 65) or may not (66, 67) be related to CD23. Ligelizumab performs better than omalizumab in inhibition of IgE synthesis, reportedly due to its engagement with CD23-bound IgE, forming aggregate complexes that omalizumab is not involved with (35). Yet, the same interaction with IgE-CD23 complexes has been cited to possibly mimic IgE-allergen complexes that triggered eosinophilic lung inflammation in the treatment of allergic asthma (35, 68). Whether this truly occurs with ligelizumab or is applicable to other anti-IgE mAbs remains to be elucidated.

Presently, UB-221 is being studied for treating CSU as its first clinical indication. A single i.v. infusion of UB-221 has shown clinical efficacy in patients with CSU, with disease symptom relief expressed as a dose-dependent decrease in UAS7 score (Figure 9 and Supplemental Figure 8A). Administration of UB-221 has exhibited a pattern of durable suppression of serum free IgE and slow return to the baseline IgE level (Supplemental Figure 8B), and a parallel rapid reduction in FcεRI on basophils (Supplemental Figure 8C). In addition to potent IgE neutralization, the real-world impact of significant CD23-mediated IgE downregulation by UB-221 (presumably via an increase in IgE clearance and inhibition of IgE neosynthesis), if the IgE downregulation is manifested in the clinic, would become apparent only when administered under repeat dosing for a substantially long period of time.

With regard to safety, UB-221’s interaction with IgE-CD23 does not trigger antibody-dependent cell-mediated cytotoxicity (ADCC) or complement-dependent cytotoxicity (CDC) activities, as shown in CD23-overexpressing SKW6.4 B lymphoma cells preloaded with IgE (Supplemental Figure 10). This suggests that oligomerization of UB-221–IgE and UB-221–IgE-CD23 complexes (Figures 2 and 3 and Supplemental Figure 9) may not present a safety issue. The presence of UB-221 does not activate human primary basophils (Figure 1A). In the IND-enabling safety/toxicity study in cynomolgus macaques, UB-221 was safe and well tolerated following 4 weekly i.v. doses or 16 weekly s.c. doses up to 100 mg/kg. No significant cytokine release was observed in human PBMCs treated with UB-221. A single dose of UB-221 up to 10 mg/kg did not produce any serious adverse event in the phase I clinical trial (Supplemental Table 4).

We have demonstrated that UB-221 induced a rapid, pronounced reduction in serum IgE after only a single i.v. dose in cynomolgus macaques and hIGHE-knockin mice (Figure 8), where UB-221 has been shown to be safe and well tolerated. Clinical efficacy after a single dose of UB-221 has been confirmed in patients with CSU, with disease symptom relief in parallel with rapid reduction in both serum free IgE and FcεRI on basophils.

Altogether, the unique characteristics of UB-221, a combined potent IgE neutralizer and a significant CD23-mediated IgE synthesis downregulator, has translated into favorable clinical outcome in the phase I single-dose trial with CSU patients (ClinicalTrials.gov NCT03632291) that has demonstrated durable disease symptom relief (Figure 9). That UB-221 retains free interaction with CD23 warrants further clinical research to elucidate its therapeutic implications.

## Methods

### Recombinant anti-IgE mAbs and proteins

UB-221 derived from the parental 8D6 murine mAb was generated by CDR grafting as described in the Supplemental Methods and produced in CHO-S cells at UBP. Omalizumab (Xolair) was from Novartis. Ligelizumab, constructed according to the published amino acid sequence (https://drugs.ncats.io/drug/L8LE0L691T), was obtained from Creative Biolabs (QGE031, catalog no. TAB-755), or produced in-house at UBP in stably transfected CHO-S cells.

The proteins used in key bioassays, human FcεRIα-Fcγ and trimeric CD23 (ectodomain), were obtained from Academia Sinica, Taiwan. Expi293 cells were from Thermo Fisher Scientific. Human full-length IgE was purified from the culture medium of U266 myeloma cells. Cynomolgus macaque IgE Fc fragment (cIgE) and trimeric CD23 ectodomain (cCD23) were constructed in-house and expressed in Expi293 cells.

### Cell lines

The RBL SX-38 cell line, engineered to express human FcεRI on rat basophilic leukemia cell membranes, was licensed from Harvard University. The B cell lymphoma Ramos cells stably expressing mIgE Fc_L_ (the Fc portion of a membrane-bound long form of human IgE) was obtained from Academia Sinica, Taiwan. The B cell lymphoma SKW6.4 cell line was from ATCC.

### Cynomolgus monkeys and hGHE-knockin mice

Cynomolgus monkeys used to evaluate the effect of UB-221 (a single i.v. dose at 5 mg/kg) on serum IgE reduction was conducted in JOINN Laboratories. The hGHE-knockin mouse strain was licensed from Academia Sinica, Taiwan. The original hIGHE mouse strain F0 (male C57BL/6 background) was crossbred with female BALB/c mice to generate the F1 hybrid mice. The human IgE levels were evaluated in 108 F1 hybrid mice between 8 and 16 week old, and mice with higher human IgE levels were selected for further studies. The mice were administered i.p. with 0.3 or 3.0 mg/kg UB-221 to test the potency of serum IgE neutralization. ELISAs of serum IgE concentrations in monkeys and mice are detailed in the Supplemental Methods.

### Formation of UB-221–IgE immune complexes measured by FDS-AUC

The study of UB-221–IgE complexes formed in PBS solution and human serum was conducted at U-Medico, using FDS-AUC that enables the analysis of crowded solutions where absorbing molecules other than target protein are present, for which UB-221 mAb was labeled with Alexa Fluor 488 dye (Molecular Probes, A20100). The experimental procedures are detailed in Supplemental Methods.

### IgE protein and IgE mRNA expression in PBMCs treated with UB-221, ligelizumab, and omalizumab

The comparative PBMC IgE study was conducted for UB-221, ligelizumab (UBP lot), and omalizumab. PBMCs from fresh whole blood of 3–5 healthy donors were isolated and cultured with procedures as described above. The cells were stimulated with the anti-CD40 antibody (G28-5) (LifeSpan BioSciences) and IL-4 (R&D Systems) at a final concentration of 100 ng/mL for 11 days in the presence of 1, 3, 10, 20, or 80 μg/mL anti-IgE antibodies. Unstimulated, untreated, and human IgG antibody–treated cells served as controls. The supernatants were assayed for total IgE, IgM, and IgA by ELISA as detailed in the Supplemental Methods.

The PBMCs from 5 healthy donors were used for the study of effects on IgE mRNA expression by UB-221, ligelizumab (UBP lot), and omalizumab. The procedures were identical to the methods described above except the cell numbers of PBMCs (4 × 10^6^ cells/mL/well) and anti-IgE antibody concentrations (1 and 20 μg/mL). Total mRNA was isolated using a Direct-zol RNA Miniprep Plus Kit (Zymo Research). Isolated mRNA was reverse transcribed using SuperScript IV VILO Master Mix (Invitrogen). The qPCR analysis was performed using Fast SYBR Green Master Mix (Applied Biosystems) on a QuantStudio 5 Real-Time PCR System (Applied Biosystems). Expression was normalized to the endogenous control β2-microglobulin, and the relative expression was determined on QuantStudio Design & Analysis Software (Applied Biosystems). The sequences of the PCR primers for β2-microglobulin and IgE purchased from Integrated DNA Technologies were 5′-CACCCCCACTGAAAAAGATGAG-3′ (β2-microglobulin forward), 5′-CCTCCATGATGCTGCTTACATG-3′ (β2-microglobulin reverse), 5′-CCGTGTGGCACACACTC-3′ (IgE forward), and 5′-GTCGCAGGACGACTGTAAG-3′ (IgE reverse).

### Free IgE in sera from patients with AD in ex vivo IgE neutralization study

Serum samples collected from 30 AD patients were grouped into 3 different ranges of basal IgE levels. Serum samples in each basal-IgE group (low, <4,800; medium, 4,800–24,000; and high, >24,000 ng/mL) were incubated for 1 hour at room temperature with UB-221, ligelizumab (QGE031 from Creative Biolabs, TAB-755), or omalizumab at 1 of 3 increasing concentration ranges: 0–4,000 ng/mL for the low IgE range, 0–20,000 ng/mL for the medium IgE range, and 0–75,000 ng/mL for the high IgE range. The remaining free IgE concentrations in sera were quantitated on 96-well ELISA plates coated with FcεRIα-Fcγ. Serially diluted IgE calibration standards at 3.13 to 200 ng/mL prepared in IgE-depleted pool human serum and AD serum samples at appropriate dilution were added to the plate wells. After incubation, the plates were washed and incubated with biotin-conjugated mouse anti–human IgE mAb (BD Biosciences), followed by detection with streptavidin-poly HRP (Thermo Fisher Scientific/Pierce). The color developed with TMB substrate solution was measured at 450 nm.

### Binding of mAbs to the IgE preabsorbed on CD23+ SKW6.4 cells

The binding of anti-IgE mAbs in free form to the CD23-bound IgE on SKW6.4 cells was investigated by flow cytometry. CD23^+^ SKW6.4 cells (2 × 10^5^ cells/well) were suspended in 100 μL of PBS containing 15 μg/mL human IgE and incubated for 30 minutes at 4°C, followed by washing twice with PBS to remove unbound IgE, and then resuspended in 100 μL PBS containing UB-221, ligelizumab (UBP lot), or omalizumab at the range of 0–10 μg/mL, and incubated for 30 minutes at 4°C. The cells were washed and incubated with 100 μL PBS containing Brilliant Violet 421–conjugated anti–human IgG Fc (BioLegend, catalog no. 409318) for 30 minutes at 4°C. The cells were then washed twice and resuspended in a 400 μL fixation buffer at 4°C. The samples were analyzed on a BD FACSVerse instrument.

### Binding of mAb-IgE complexes to CD23+ SKW6.4 cells

The binding of anti-IgE mAbs in IgE-complexed form to SKW6.4 cells was analyzed by flow cytometry. First, UB-221, ligelizumab (UBP lot), or omalizumab was conjugated with Alexa Fluor 647 using an Alexa Fluor 647 antibody labeling kit (Life Technologies). An equal volume of 15 μg/mL human IgE and anti-IgE drugs at 0 to 10 μg/mL were mixed in PBS and incubated for 1 hour at room temperature. The mixtures were added to SKW6.4 cells (2 × 10^5^ cells/well) and incubated for 30 minutes at 4°C. The cells were then washed twice and resuspended in a 400 μL fixation buffer at 4°C. The next day, samples were analyzed on the BD FACSVerse.

### A single UB-221 dose to patients with CSU for evaluation of safety and efficacy

A phase I, open-label, dose-escalation trial to evaluate the safety, tolerability, pharmacokinetics, and pharmacodynamics of a single i.v. infusion dose of UB-221 as an add-on therapy was conducted in patients with CSU under the first-line H1-antihistamine treatment (ClinicalTrials.gov NCT03632291). The study participants in 5 dose cohorts (0.2, 0.6, 2.0, 6.0, or 10 mg/kg; *n =* 3/cohort, total 15 study participants) were monitored for 14 weeks after dosing. Full details of the trial design, inclusion and exclusion criteria, conduct, oversight, and statistical analyses are available in the Study Protocol in the Supplemental Appendix. Laboratory assay of serum concentrations of UB-221, free IgE, and FcεRI are provided in the Supplemental Methods.

### Statistics

#### Preclinical studies.

IgE protein production and mRNA expression (Figure 4) and neosynthesis of IgE, IgA, and IgM (Supplemental Figure 6) between treated and untreated groups in human PBMCs were estimated using 2-way ANOVA with Tukey’s multiple comparison test referenced to untreated controls. Comparison of mAb drug concentrations to achieve a reduction of serum IgE by 90% (EC_90_) of the basal IgE levels between treated groups (Figure 7B) were estimated by the Kruskal-Wallis 1-way ANOVA on ranks followed by Tukey’s multiple comparison test. All statistical tests were performed using SigmaPlot software (version 13.0) and were 2-sided with significance level of 0.05.

#### Clinical trial.

The intent-to-treat population, defined as all subjects who received any amount of UB-221, was used for safety, pharmacokinetics, pharmacodynamics, and efficacy analyses. IgE levels and UAS7/HSS7 scores were summarized using descriptive statistics. UAS7 score changes from baseline were estimated by the paired *t* test or Wilcoxon’s signed-rank test (for differences not normally distributed). The statistical tests were conducted using SAS software (version 9.3) and were 2-sided with a significance level of 0.05. The tests were not adjusted for multiplicity since the statistical analysis plan did not include correcting for multiplicity in this trial.

### Study approval

#### Animal studies.

The monkey study was conducted in JOINN Laboratories (Suzhou, China), which was fully accredited by the Association for Assessment and Accreditation of Laboratory Animal Care International (AAALAC). The certificate number of the animal facility is SYXK (Su) 2011-0029, and the IACUC number for this study is ACU15-542. The mouse study was conducted in United BioMedical, Inc., Asia (Zhubei, Hsinchu County, Taiwan). The IACUC number for this study is AT1502.

#### Clinical trial.

This trial was designed and sponsored by UBP and was conducted according to Good Clinical Practice at 2 sites in Taiwan. The Institutional Review Board at National Taiwan University Hospital in Taipei (IRB number 201806085MSD) and Kaohsiung Chang Gung Memorial Hospital in Kaohsiung (IRB number 201901519A0) reviewed and approved the protocol. All participants provided written informed consent before receiving any of the trial procedures.

## Author contributions

BSK, CH Li, and JBC designed and managed the preclinical research and IND-enabling studies. BSK, CH Li, and JBC contributed equally to this article. BSK, JBC, MJL, and CYW cowrote the manuscript. YYS managed the FDS-AUC based study of UB-221–IgE complex formation and conducted SPR assays and ELISAs. YJL oversaw production of UB-221 and research tool proteins. YJL and JHK were responsible for the workup of 3D structure illustrations for the mAb-IgE, FcεR1α-IgE, and mAb-IgE-CD23 molecular interfacing. YFH and MSW were responsible for method development and quantitative assay of free IgE in buffer solution and serum-associated IgE-neutralization studies. JBC, YNC, and PHT contributed to PBMC studies of de novo IgE protein and mRNA syntheses. HXH and MCL contributed to apoptosis, ELISA-, and FACS-based IgE-CD23 binding studies. JBC, YYS, HKC, and YJW contributed to the basophil activation and degranulation studies. TC performed ADCC and CDC experiments. For the clinical phase I trial, CYC and CH Lee as the clinical Principal Investigators and CH, HWS, YFL, AL, JY, and CYL from the Medical and Clinical Affairs teams contributed to the implementation of the clinical studies, and acquired and interpreted the clinical data; and FHY, CH Lin, and WT were responsible for assay development and validation, laboratory testing and data collection, and preparation of respective reports. SL, MJL, and CYW were responsible for the anti-IgE mAb clinical development plan for IgE-mediated allergic diseases. All other authors contributed to the implementation of the study and data collection. All authors critically reviewed and approved the final version. CYW had final responsibility for the decision to submit the manuscript for publication.

## Supplementary Material

Supplemental data

## Version 1 08/01/2022

Electronic publication

## Version 2 02/07/2023

Correct the spelling of author's name, Fu-Hung Yang
