# Supplementary material for: IgE-neutralizing UB-221 mAb, distinct from omalizumab and ligelizumab, exhibits CD23-mediated IgE downregulation and relieves urticaria symptoms
Source: J Clin Invest. 2022 Aug 1;132(15):e157765. doi: 10.1172/JCI157765 (PMC9337824; doi:10.1172/JCI157765)
Supplement: Supplemental data [file jci-132-157765-s011.pdf]

SUPPLEMENTAL MATERIAL

Supplement to:

Kuo B-S, Li C-H, Chen J-B, et al. ***IgE-neutralizing UB-221 mAb, distinct from omalizumab and ligelizumab, exhibits CD23-mediated IgE-downregulation and relieves urticaria symptoms***

Supplementary Figures

Fig. S1. Humanization of 8D6 ……………………………………………………………3

Fig. S2. UB-221 does not bind to the FcεRI-bound IgE on ELISA……………………….4

Fig. S3. UB-221 does not bind RBL XL-38 cells to trigger allergic degranulation……… 5

Fig. S4. UB-221 can interact with IgE:CD23 but omalizumab cannot…………………... 6

Fig. S5. Time-dependent IgE protein synthesis in human PBMCs of healthy volunteers...7

Fig. S6. Effects on neo-syntheses of IgE, IgA and IgM in human PBMCs by UB-221 vs. omalizumab…………………………………………………………………8

Fig. S7. IgE-neutralizing potency on ELISA of anti-IgE mAbs in PBS buffer solution…. 9

Fig. S8. Concurrent changes of UAS7 disease score, serum free IgE level and FcεRI expression on basophils in individual CSU patients after a single IV infusion of UB-221…………………………………………………………………….. 10

Fig. S9. Superposed 3D structures of mAb:IgE:CD23 complexes to differentiate the spatial conformations upon the interaction of three anti-IgE mAbs with CD23…11

Fig. S10. UB-221 does not induce CDC and ADCC effects in CD23+-SKW6.4 B cells….. 12

Supplementary Tables

Table S1. %Reduction of IgE protein produced in human PBMCs incubated with UB-221, ligelizumab, or omalizumab………………………………………..13

Table S2. %Reduction of IgE mRNA produced in human PBMCs incubated with UB-221, ligelizumab, or omalizumab………………………………………..14

Table S3. Baseline demographics and clinical characteristics of study participants with chronic spontaneous urticaria……………………………………………….. 15

Table S4. Adverse events developed after a single IV infusion of UB-221………………16

Table S5. Pharmacokinetic parameters after a single IV infusion of UB-221……………..17

Supplementary Methods

- Binding to the FcεRI-bound IgE on ELISA……………………………………………18
- Binding to FcεRI-bound IgE on ELISA…………………………………………………. 18
- Binding to IgE on RBL SX-38 basophils………………………………………………18
- Binding of UB-211:IgE complex to and activation of rat huFcεRI+-RBL SX-38 cells…..18
- Binding of UB-221:IgE complex to CD23+-SKW cells…………………………………19

▪ Binding of anti-IgE mAbs to CD23-bound IgE on ELISA……………………………….19
▪ Competitive inhibition of IgE binding to FcεRI by UB-221 vs. ligelizumab on ELISA... 19
▪ IgE binding affinity on SPR…………………………………………………………19
▪ RBL SX-38 degranulation assay……………………………………………………20
▪ Competitive inhibition of IgE-binding to FcεRI on RBL SX-38 cells…………………... 20
▪ Effects of UB-221-vs.-omalizumab on IgE protein production in PBMCs and ELISA assays of total IgE, IgM, and IgA……………………………………………………….. 20
▪ Binding of anti-IgE mAbs to the CD23-bound IgE…………………………………21
▪ Binding of mAb:IgE complex to CD23……………………………………………..21
▪ Free chimerized IgE in sera of hIGHE-knockin mice…………………………… 21
▪ Free IgE concentrations in sera of cynomolgus macaques after a single IV infusion dose of UB-221……………………………………………………………………21
▪ Serum UB-221 concentrations in cynomolgus macaques after a single dose of UB-221.. 22
▪ Free IgE remained in buffer solution after competitive inhibition of IgE binding to FcεRI……………………………………………………………………………..22
▪ Superposed 3D structures of mAb:IgE:CD23 complexes, mAb:IgE, and FcεRIα:IgE for anti-IgE mAbs…………………………………………………………………… 22
▪ Apoptosis to mIgE-Ramos by anti-IgE mAbs…………………………………… 23
▪ Antibody-dependent cellular cytotoxicity (ADCC) to CD23^{+}-SKW6.4 B cells…………23
▪ Complement-dependent cytotoxicity (CDC) to CD23^{+}-SKW6.4 B cells………………...23
▪ Measurement of UB-221 in sera of Phase-1 study participants…………………………..24
▪ Measurements of free IgE in sera of Phase-1 study participants………………………… 24
▪ Measurement of FcεRI expression on basophils of Phase-1 study participants………… 25
▪ Formation of UB-221:IgE immune complexes in PBS and human serum by FDS-AUC..25
▪ Binding of anti-IgE mAbs to mIgE-Ramos B-lymphoma cells…………………………..26

Supplementary Appendix

Appendix 1

Phase 1 Study A-107 protocol

Appendix 2

Phase 1 Study A-107 IRB approval letter

Appendix 3

Phase 1 Study A-107 Informed Consent Form (ICF)

Supplementary Figures

Figure S1. Humanization of 8D6

A

```
            1             10             20             30             40             50
VH1-69/JH4  QVQLVQSGAEVKKPGSSVKVSCKASGGTFSSYAISWVRQAPGQGLEWMGG
  8D6  VH   QVQLQQSGAELAKPGASVMLSCKASGYTFNGYWMHWVKQRPGQDLEWIGY
UB-221 VH   QVQLVQSGAEVKKPGSSVKVSCKASGYTFNGYWMHWVRQAPGQGLEWIGY
                                                           *

            51a        60            70            80 abc          90
VH1-69/JH4  IIPIFGTANYAQKFQGRVTITADESTSTAYMELSSLRSEDTAVYYCAR--
  8D6 VH    INPTTGHTEYNQKFKDKATLTADESSNTAYIELSSLTSDDSAVYYCARQE
UB-221 VH   INPTTGHTEYNQKFKDKATITADESTNTAYMELSSLRSEDTAVYYCARQE
                             **        *

               100         110
VH1-69/JH4  ----YFDYWGQGTLVTVSS
  8D6 VH    YRHSWFAYWGQGTLVTVSA
UB-221 VH   YRHSWFAYWGQGTLVTVSS
```

B

```
            1            10             20       abcd 30             40
Vκ1-39/Jκ1  DIQMTQSPSSLSASVGDRVTITCRASQSIS----SYLNWYQQKPGKAPKL
  8D6 Vκ    DIVLTQSPASLAVSLGQRATISCKASQSVDYDGDTYMNWYHQKPGQPPKL
UB-221 Vκ   DIQLTQSPSSLSASVGDRVTITCRASQSVDYDGDTYMNWYQQKPGKAPKL
               *

               50            60            70            80            90
Vκ1-39/Jκ1  LIYAASSLQSGVPSRFSGSGSGTDFTLTISSLQPEDFATYYCQQS-YSTW
  8D6 Vκ    LIYAASNLDSGIPARFSGSGSGTDFTLNIHPVEEEDAATYYCQQTNEDPW
UB-221 Vκ   LIYAASNLDSGVPSRFSGSGSGTDFTLTISSLQPEDFATYYCQQTNEDPW

               100      107
Vκ1-39/Jκ1  TFGQGTKVEIKR
  8D6 Vκ    TFGGGTKLEIKR
UB-221 Vκ   TFGQGTKVEIKR
```

(A) The alignment of amino acid sequences of variable heavy chains including human germline VH1-69 and JH4 sequences (VH1-69/JH4), murine anti-IgE antibody 8D6 (8D6), and UB-221 (humanized 8D6). **(B)** The alignment of amino acid sequences of variable light chains including human germline Vκ1-39 and Jκ1 sequences (Vκ1-39/Jκ1), 8D6, and UB-221 (humanized 8D6). Complementarity determining regions (CDRs) are underlined. The amino acids of the murine VH and Vk framework retained in UB-221 are labeled by stars.

Figure S2. UB-221 does not bind to the FcεRI-bound IgE on ELISA

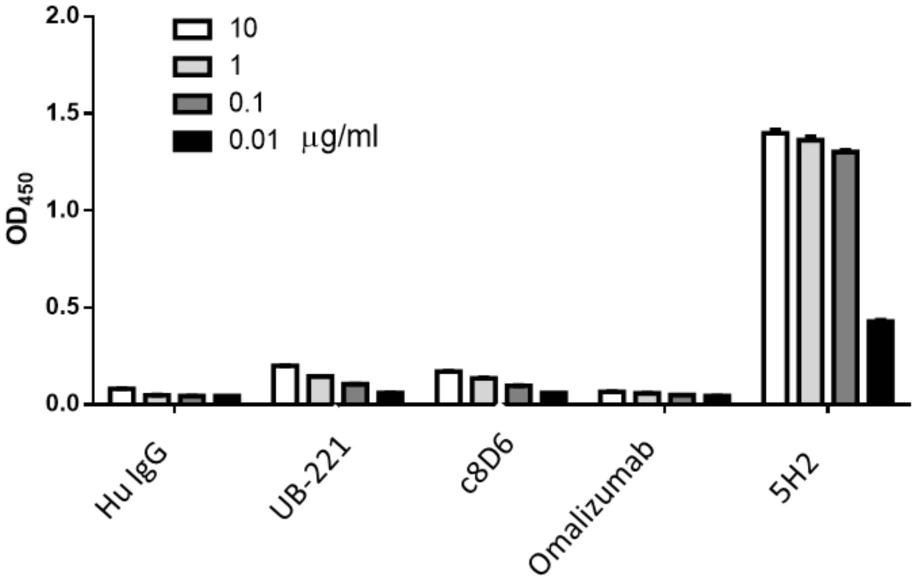

A representative ELISA assay, where the human IgE was absorbed to the plate-immobilized FcεRI, to show no significant IgE binding by UB-221, c8D6 (human-murine chimerized version of UB-221), omalizumab, and human IgG (negative control) at the indicated concentration range. The 5H2, a mouse anti-human IgE mAb (AbD Serotec) was used as a positive control that caused the strong binding to the FcεRI-bound IgE.

Figure S3. UB-221 does not bind RBL XL-38 cells to trigger allergic degranulation

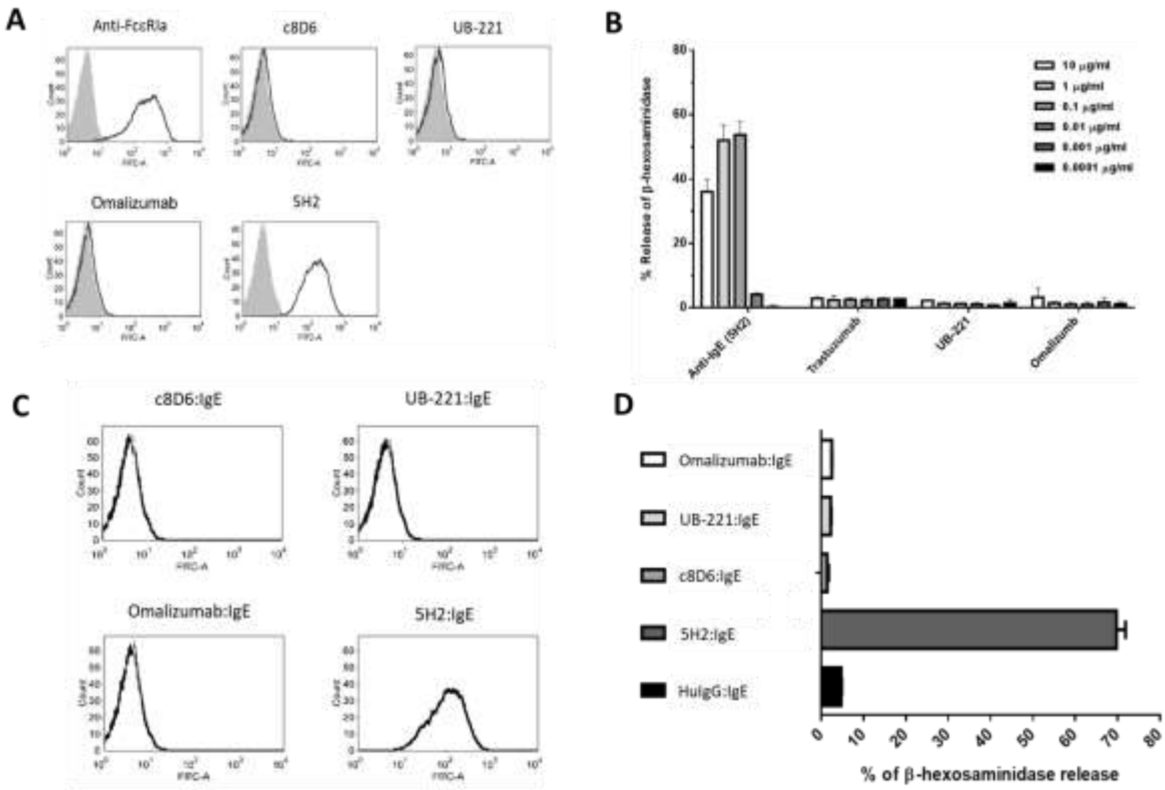

RBL SX-38 is a rat basophilic leukemia cell line expressing the α, β, and γ chains of human FcεRI. UB-221, c8D6 (human-murine chimerized version of UB-221), and omalizumab were investigated in the cellular binding by flow cytometry and degranulation by assay of β-hexosaminidase release, where human IgG or trastuzumab (anti-her2 mAb) was used as a negative control, and murine anti-IgE mAb 5H2 or anti-FcεRI mAb was used as a positive control. **(A)** On FcεRI^{+}-RBL SX-38 cells with human IgE pre-loaded, UB-221, c8D6, and omalizumab in a free form did not bind to the cells, while 5H2 and anti-FcεRI did. **(B)** On IgE-preloaded FcεRI^{+}-RBL SX-38 cells, UB-221, trastuzumab, and omalizumab in a free form did not activate the cells to degranulate, while 5H2 did. **(C)** In a pre-formed IgE immune complex at a 1:1 molar ratio, c8D6:IgE, UB-221:IgE, and omalizumab:IgE did not bind to the FcεRI^{+}-RBL SX-38 cells and **(D)** so did not activate the cells to degranulate, while 5H2:IgE did bind the cells and activate degranulation.

Figure S4. UB-221 can interact with IgE:CD23 but omalizumab cannot.

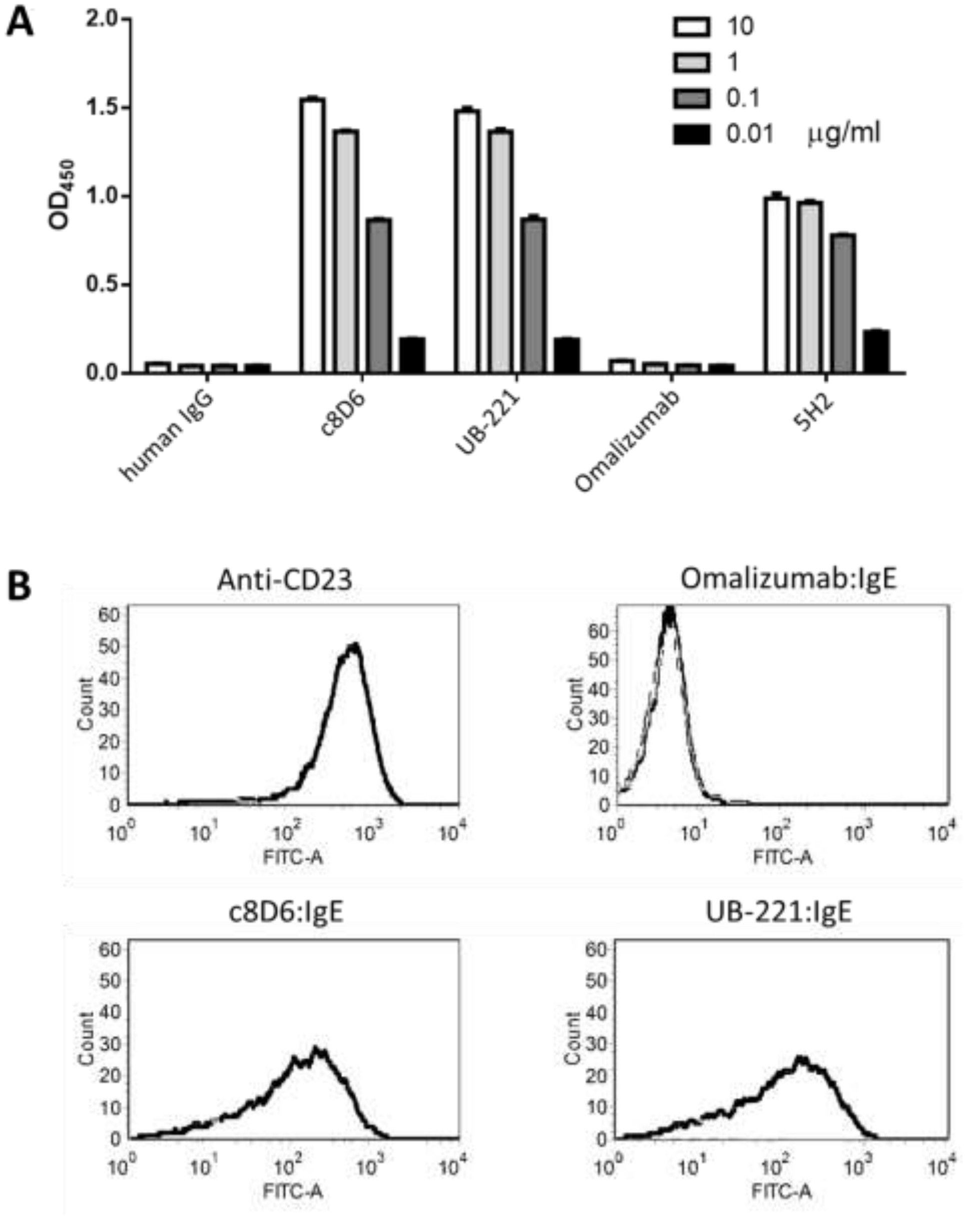

Interaction with IgE:CD23 can influence the CD23-mediated functions including regulation of IgE production. **(A)** On ELISA with recombinant human CD23 immobilized and IgE pre-absorbed, UB-221, c8D6 (the human murine chimerized version of UB-221), and 5H2 (a murine anti-IgE mAb as positive control) were found to bind dose-dependently to the IgE, while omalizumab and human IgG (negative control) did not. **(B)** Analyzed on flow cytometry with CD23-expressing SKW6.4 cells, anti-CD23 mAb (positive control), and UB-221 and c8D6 in a pre-formed complex (UB-221:IgE and c8D6:IgE) at 1:1 molar ratio were shown to bind to the cells, while the omalizumab:IgE complex did not interact with CD23.

Figure. S5. Time-dependent IgE protein synthesis in human PBMCs of healthy volunteers

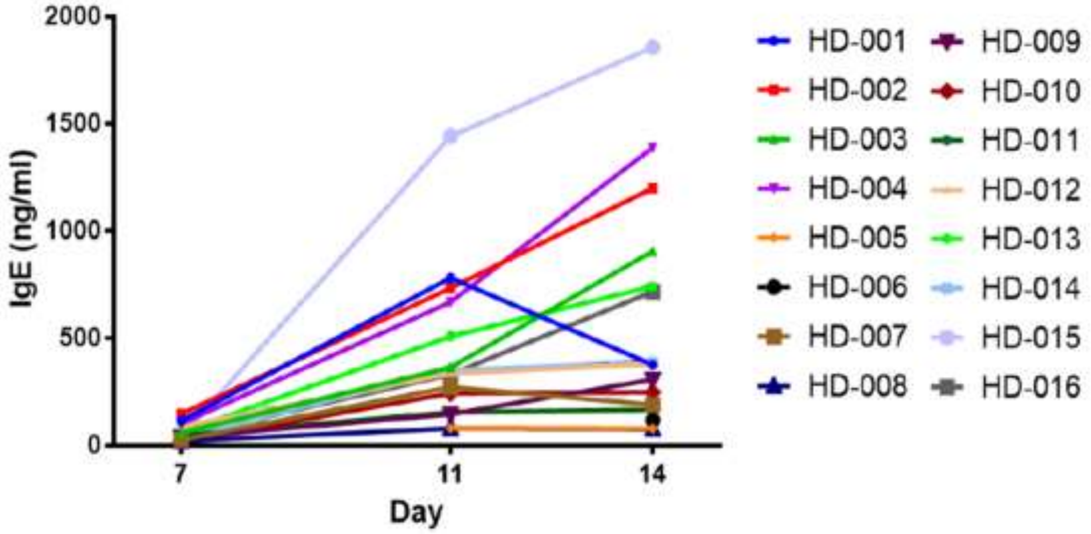

A study model of IgE neo-synthesis in PBMCs was conducted using fresh blood of healthy donors (HD-001 to HD-016) under co-stimulation with IL-4 and an anti-40 antibody. The PBMCs isolated from blood samples were incubated in a serum-free culture medium for indicated days and the detected IgE represents a *de novo* IgE production derived from the stimulated IgE-producing B cells. The study model exhibits a time-dependent, individual-dependent IgE increase starting from Day 7 that plateaued at Days 11-14. IgE was normally detectable about one week after the start of incubation. The IgE concentrations fell between 79.8 ng/ml to 1856 ng/mL, which increased 3.5 to 45 folds in comparison to the IgE concentrations measured on day 7. The levels of total IgE were assayed on ELISA as a measure reflecting the IgE neo-synthesis started from Day 0. There was an apparent between-individual difference in the level of IgE production. The method was established for comparative effects of anti-IgE agents on the IgE synthesis, in addition to IgE neutralization.

Figure S6. Effects on neo-syntheses of IgE, IgA and IgM in human PBMCs by UB-221 vs. omalizumab

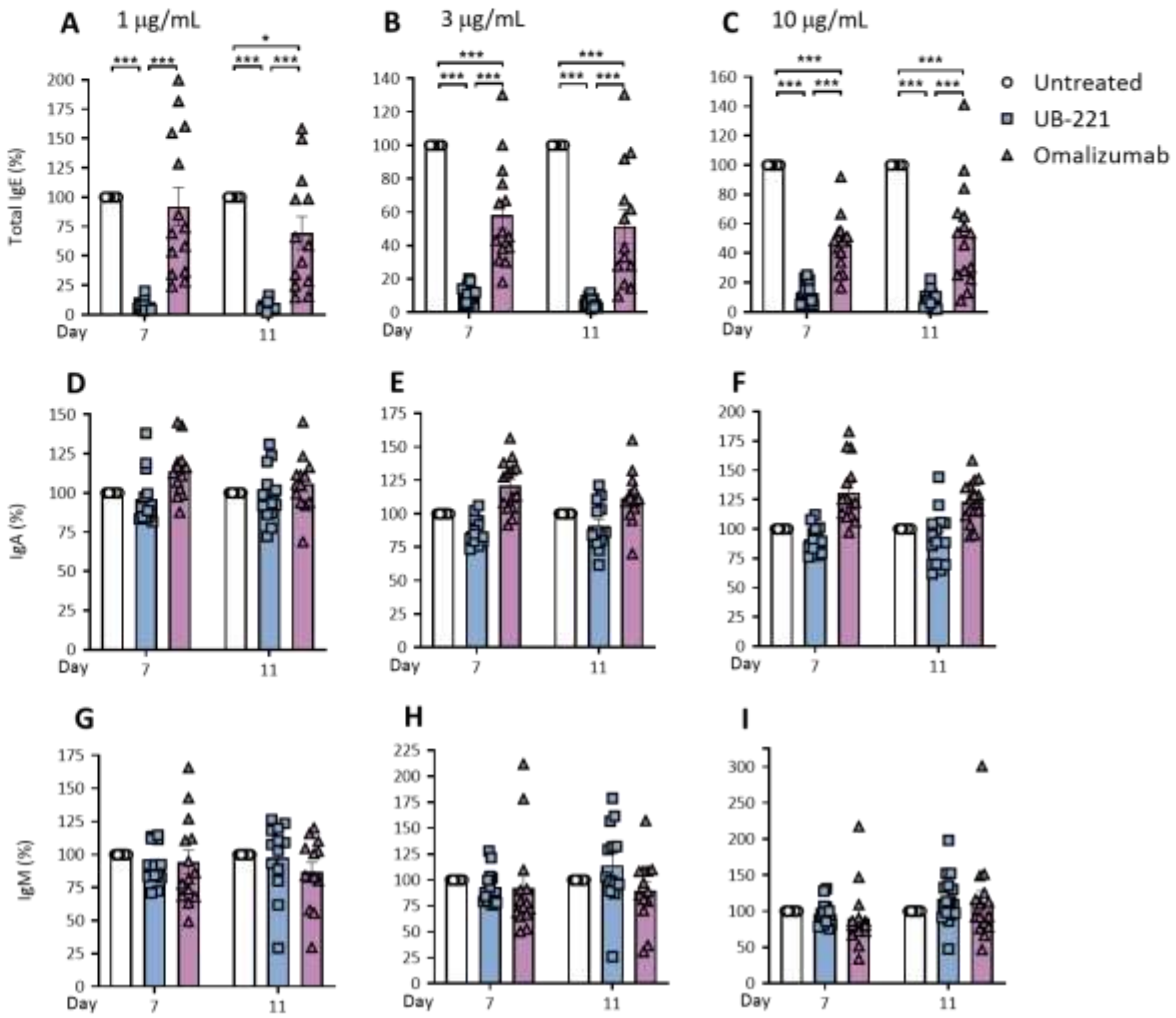

In the presence of UB-221 or omalizumab, human PBMCs from 14 healthy donors were stimulated with recombinant human IL-4 and an anti-human CD40 antibody to undergo *de novo* IgE synthesis and assayed on Days 7 and 11. The effects on the production of IgA and IgM were also investigated. The comparative effects of the two anti-IgE mAbs on IgE production are shown for the doses at **(A)** 1 μg/mL, **(B)** 3 μg/mL, and **(C)** 10 μg/mL. The correspondent IgA productions are presented in **(D)-(F)** and IgM production in **(G)-(I)**. The total IgE, IgA, and IgM in cell culture supernatant samples were quantified by ELISA. The productions of IgE were affected by the anti-IgE mAbs, however, IgA and IgM were not affected. The calculated percentages of IgE reduction are shown in Supplementary Table 1a, which were obtained using IgE levels of the respective untreated cells set as 100%. Data are shown as Mean±SEM. Different treatments were compared relative to the untreated group using two-way ANOVA with Tukey's multiple comparison referenced to untreated controls. *$P < 0.05$, **$P < 0.01$, ***$P < 0.001$.

Figure. S7. IgE-neutralizing potency on ELISA of anti-IgE mAbs in PBS buffer solution

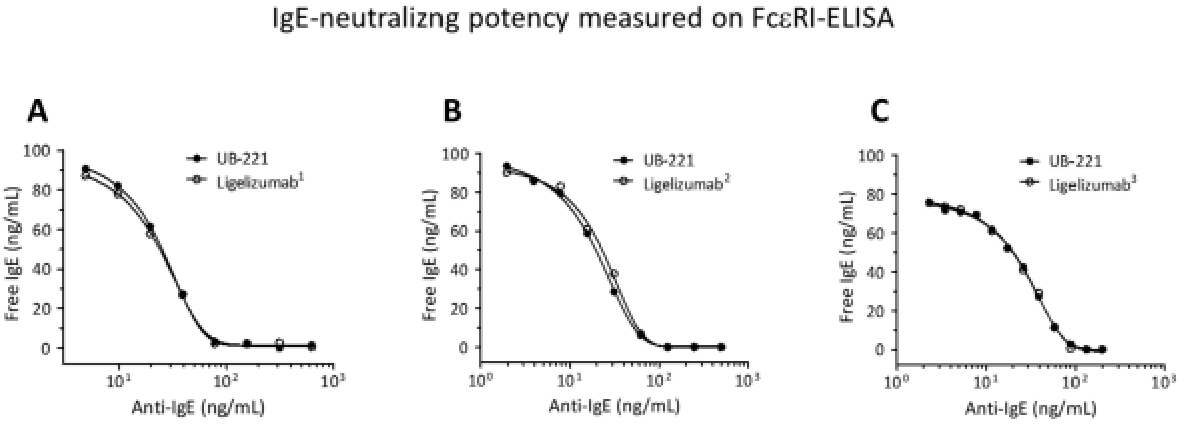

On FcεRI-immobilized ELISA, competitive neutralization of IgE binding by UB-221 and ligelizumab were compared on three separate occasions **(A)** ligelizumab[1] from Creative Biolabs (CN: TAB755); **(B)** ligelizumab[2] and **(C)** ligelizumab[3] from UBP in-house Lot 040620 and Lot 090320, respectively. The neutralizing activities were expressed as the decreasing presence of free, unbound IgE that remained in the solution phase. The neutralization curves of the two comparative antibodies (UB-221 vs. ligelizumab) in each of all test occasions are superimposable and equipotent with overall IC_{50} values in the range of 21-26 ng/mL.

Figure. S8. Concurrent changes of UAS7 disease score, serum free IgE level and FcεRI expression on basophils in individual CSU patients after a single IV infusion of UB-221

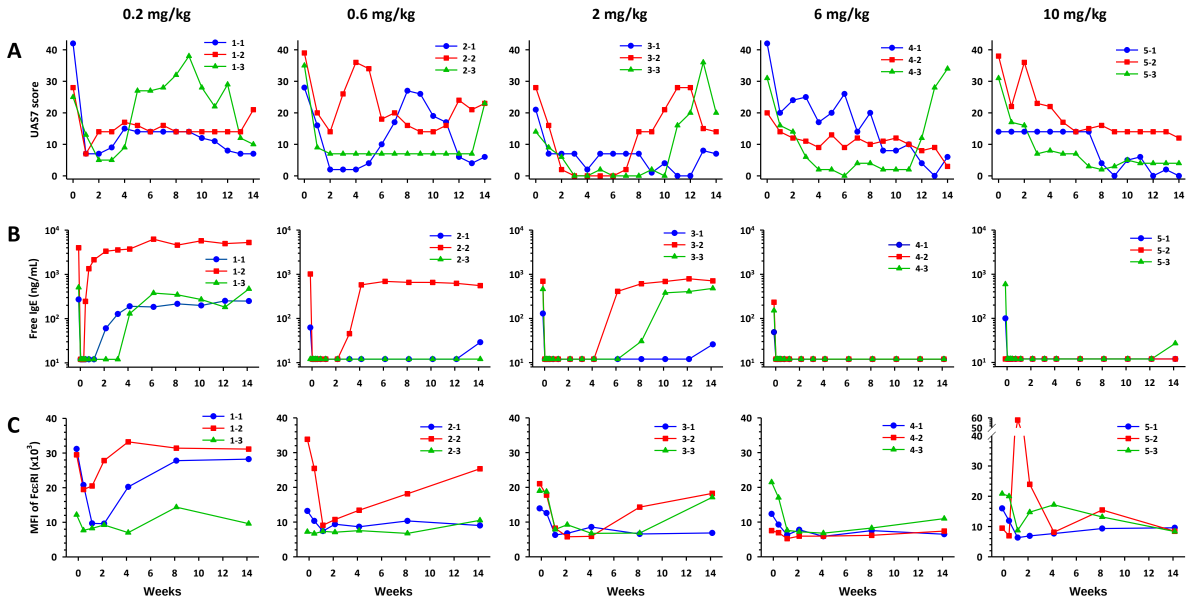

Shown in parallel are the individual study participant's **(A)** UAS7 disease symptom score, **(B)** serum free IgE concentration, and FcεRI level on basophils of CSU patietns in 5 dose cohorts (0.2 to 10 mg/kg ; n = 3 per dose cohort) over 14 weeks of the Phase-1 single IV UB-221 dose trial. Of 15 study participants, 13 were defined having moderate to severe CSU at baseline (UAS7 score≧16) and 2 had mild CSU. The individual profiles illustrate the dose- and time-dependent changes in the three biomarkers of UAS7, serum free IgE, and basophilc FcεRI (expressed as MFI on flow cytometry). Reduction of UAS7 correlates with decreases in both serum free IgE and FcεRI on basophils.

Figure. S9. **Superposed 3D structures of mAb:IgE:CD23 complexes to differentiate the spatial conformations upon the interaction of three anti-IgE mAbs with CD23**

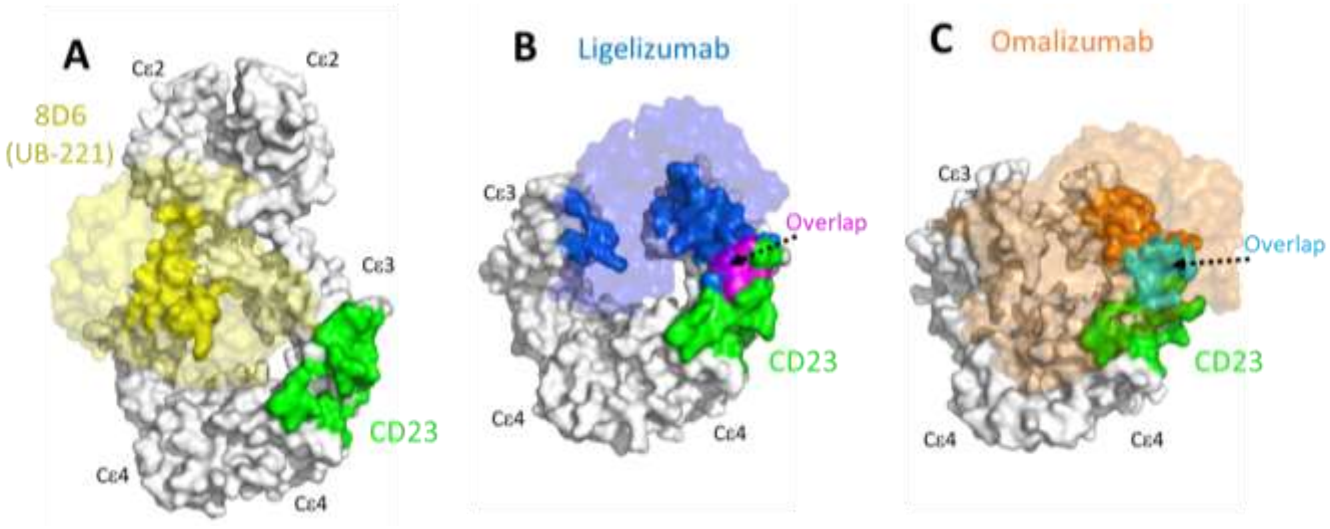

The 3D illustrations of the superposed mAb:IgE:CD23 complex structure are generated using the Protein Data Bank accession number 6EYO (8D6:IgE), 6UQR (ligelizumab:IgE), 5HYS (omalizumab:IgE) and 4EZM (IgE:CD23). The structure presentations were generated using the PyMOL program. The IgE:CD23 complex reveals that CD23 binds to the epitopes at the juncture of Cε3-Cε4 of IgE molecule and these contact residues on IgE (gray) are shown in green. **(A)** The Fab of 8D6 can bind to the site on IgE (yellow) distant from the site where IgE and CD23 contact (green), without overlapping; the surface representation of the 8D6 Fab binding (transparent yellow) does not present a steric conflict with the IgE:CD23 interaction zone. **(B)** Ligelizumab (blue) binds the sites on IgE at a location that partially overlaps the sites where IgE and CD23 interact (magenta); the surface representation of the ligelizumab binding (transparent blue) presents a minor steric conflict with the IgE:CD23 interaction zone. **(C)** Omalizumab (brawn) binds IgE at sites on IgE that substantially overlap the sites where IgE and CD23 interact (cyan); the surface representation of the omalizumab binding (transparent brawn) presents a steric conflict with the IgE:CD23 interaction zone that is wider than that by ligelizumab. The atomic volumes of the overlapping (magenta and cyan) had been calculated and shown to be a 100-fold difference, approximately 28 for ligelizumab vs. 2773 for omalizumab (Gasser P, et al., 2020, doi:10.1038/s41467-019-13815-w).

Figure. S10. UB-221 does not induce CDC and ADCC effects in CD23^{+}-SKW6.4 B cells

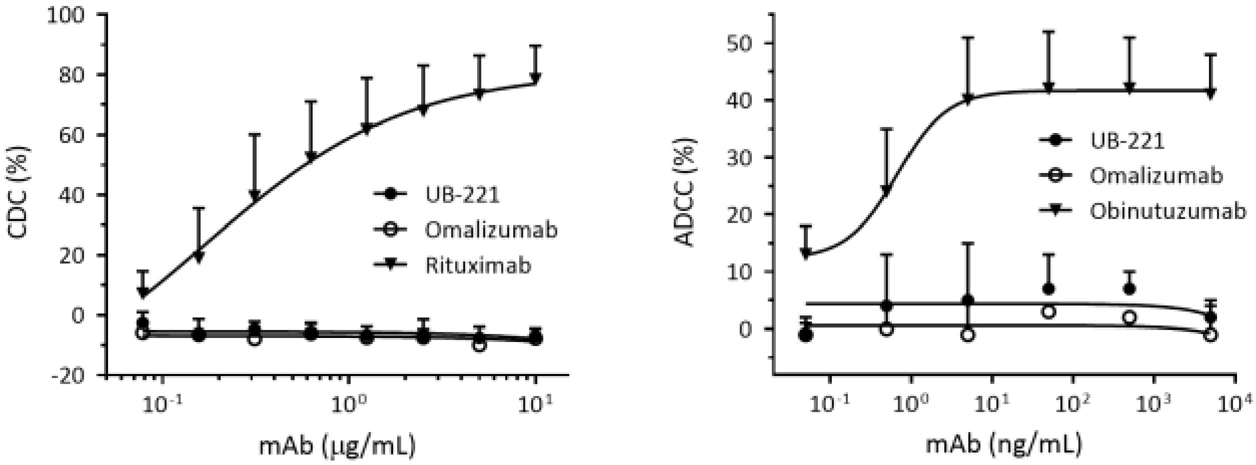

CDC (Complement-dependent cytotoxicity) effects (%CDC, Mean±SD, n = 3) induced by rituximab (positive control), UB-221, and omalizumab (negative control) was investigated in human B lymphoma CD20^{+}CD23^{+}-SKW6.4 cells preabsorbed with human IgE, incubated with 20% human serum, using rituximab (anti-CD20) was a positive control and omalizumab was a negative control. ADCC (antibody-dependent cellular cytotoxicity) effects (%ADCC, Mean±SD, n = 3) induced by the anti-CD20 obinutuzumab as a positive control, UB-221, and omalizumab as a negative control was investigated with human B cell lymphoma CD23-overexpressed SKW 6.4 cells as the target cells, which were preabsorbed with human IgE and human PBMC cells as the effector cells. In CDC and ADCC tests, the reaction mixtures were incubated with tested monoclonal antibodies; the anti-IgE mAbs would bind to the CD23-bound IgE. The results show no CDC or ADCC effect induced by UB-221.

Supplementary Tables

Table S1. %Reduction of IgE protein produced in human PBMCs incubated with UB-221, ligelizumab[1], or omalizumab

	mAb	Conc.[2] (μg/mL)	Day 7 (%)	Day 11 (%)	Day 14 (%)
A	UB-221	1	-92.5 ± 1.2	-93.5 ± 1.1	-89.5 ± 1.7
		3	-89.6 ± 1.5	-94.1 ± 0.9	-93.1 ± 1.1
		10	-87.0 ± 2.0	-90.3 ± 1.5	-90.1 ± 1.6
	Omalizumab	1	-7.9 ± 16.3	-30.3 ± 13.5	-30.6 ± 12.3
		3	-41.6 ± 8.3	-48.4 ± 9.9	-34.0 ± 14.9
		10	-53.5 ± 5.1	-42.0 ± 9.1	-43.4 ± 9.8

	Day	Conc.[3] (μg/mL)	UB-221 (%)	Ligelizumab (%)	Omalizumab (%)
B	11	1	-20.5±25.3	199.4±28.3	12.8±11.8
		3	-44.2±24.3	55.1±22.5	-7.9±13.2
		10	-73.5 ± 10.0	-21.9 ± 24.5	-4.9 ± 10.9
		20	-71.2 ± 4.6	-16.3 ± 20.6	-31.1 ± 14.4
		80	-68.8 ± 5.5	-30.6 ± 15.9	-14.5 ± 15.6

[1] Ligelizumab: the ligelizumab substance was produced at United Biopharma.

[2] The PBMC cells from 11 healthy human donors (n = 11) were treated with the two antibodies at three concentrations. The %Reduction rate values in Table S1A are shown as Mean±SEM corresponding to the panels A to C of Figure 4.

[3] The PBMC cells treated with the three antibodies at 1, 3, and 10 mg/mL were from 3 healthy human donors (n = 3); the PBMC cells treated with 20 and 80 □g/mL were from 5 donors (n = 5). The %Reduction rate values in the Table S1B are shown as Mean±SEM corresponding to the panels A to E of Supplementary Figure 6.

Table S2. %Reduction of IgE mRNA produced in human PBMCs incubated with UB-221, ligelizumab[1], or omalizumab

%mRNA Reduction	Day	Conc.[2] (μg/mL)	UB-221 (%)	Ligelizumab (%)	Omalizumab (%)
	**11**	1	-73.5 ± 10.0	-21.9 ± 24.5	-4.9 ± 10.9
		20	-71.2 ± 4.6	-16.3 ± 20.6	-31.1 ± 14.4

[1] Ligelisumab: the ligelizumab substance was produced at United Biopharma.

[2] The PBMC cells from 5 healthy human donors (n = 5) were treated with the antibodies at 1 and 20 mg/mL.
The %Reduction rate values in Table S2 are shown as Mean±SEM corresponding to the panels F and G of Figure 4.

Table S3. Baseline demographics and clinical characteristics of study participants with chronic spontaneous urticaria

Characteristics[1]	0.2 mg/kg (n = 3)	0.6 mg/kg (n = 3)	2 mg/kg (n = 3)	6 mg/kg (n = 3)	10 mg/kg (n = 3)
Age, years	61.0 (2.7)	54.7 (15.6)	46.3 (7.6)	46.7 (23.5)	48.3 (23.8)
Gender					
Male, n (%)	2 (66.7)	0 (0)	2 (66.7)	1 (33.3)	3 (100)
Female, n (%)	1 (33.3)	3 (100)	1 (33.3)	2 (66.7)	0 (0)
Race: Asian, n (%)	3 (100)	3 (100)	3 (100)	3 (100)	3 (100)
BMI, kg/m^2	25.1 (4.8)	21.0 (2.1)	23.5 (4.7)	22.5 (1.8)	22.3 (1.8)
Xolair experience, n (%)[2]	0 (0)	1 (33.3)	0 (0)	1 (33.3)	0 (0)
Clinical outcome by[3]					
UAS7[4]	31.7 (9.1)	34.0 (5.6)	21.0 (7.0)	31.0 (11.0)	27.7 (12.3)
HSS7	14.3 (6.5)	18.0 (3.6)	11.7 (4.0)	14.7 (6.0)	14.3 (6.4)
ISS7	17.3 (3.5)	16.0 (3.5)	9.3 (4.0)	16.3 (5.0)	13.3 (6.0)
Baseline serum IgE (ng/mL)[5]	1592 (2087)	363 (565)	429 (285)	145 (92)	235 (313)

[1] Age, BMI, clinical outcome & serum IgE level are presented as mean (±SD). BMI, body mass index; CSU, chronic spontaneous urticaria
[2] Twos study participants experienced the last dose of Xolair ≥ 12 months prior to enrollment.
[3] Clinical outcome measurements: UAS7, weekly urticaria activity score; HSS7, weekly hives-severity score; and ISS7, weekly itch-severity score.
[4] UAS7 = HSS7 + ISS7

Table S4. Adverse events developed after receiving a single IV infusion of UB-221

Variable (%)	0.2 mg/kg		0.6 mg/kg		2 mg/kg		6 mg/kg		10 mg/kg	
	Event (N = 7)	Subject (n = 3)	Event (N = 6)	Subject (n = 3)	Event (N = 5)	Subject (n = 3)	Event (N = 14)	Subject (n = 3)	Event (N = 1)	Subject (n = 3)
Total	7 (100)	3 (100)	6 (100)	3 (100)	5 (100)	2 (66.7)	14 (100)	2 (66.7)	1 (100)	1 (33.3)
Mild	2 (28.6)	2 (66.7)	4 (66.7)	1 (33.3)	4 (80)	2 (66.7)	14 (100)	2 (66.7)	1 (100)	1 (33.3)
Moderate	5 (71.4)	3 (100)	2 (33.3)	2 (66.7)	0	0	0	0	0	0
Severe	0	0	0	0	1 (20)	1 (33.3)	0	0	0	0
AEs leading to UB-221 discontinuation	0	0	0	0	0	0	0	0	0	0
Serious AEs	0	0	0	0	0	0	0	0	0	0
AEs related to UB-221	0	0	0	0	0	0	3 (21.4)	1 (33.3)	0	0
Nausea	0	0	0	0	0	0	1 (7.1)	1 (33.3)	0	0
Vomiting	0	0	0	0	0	0	1 (7.1)	1 (33.3)	0	0
Headache	0	0	0	0	0	0	1 (7.1)	1 (33.3)	0	0

AEs, adverse events; N, number of events; n, number of participants.

Table S5. Pharmacokinetic parameters after a single IV infusion of UB-221

Parameter	0.2 mg/kg (n = 3)	0.6 mg/kg (n = 3)	2 mg/kg (n = 3)	6 mg/kg (n = 3)	10 mg/kg (n = 3)
$AUC_{0\text{-}t}$ (h*µg/mL)	519	4,160	15,800	55,800	80,400
$AUC_{0\text{-}inf}$(h*µg/mL)	549	4,290	16,000	56,600	83,800
C_{max} (µg/mL)	4.6	15.7	67.7	188	298
T_{max} (h)	6.7	2.1	3.0	2.1	3.4
$t_{1/2}$ (day)	8.3	18.3	15.0	16.6	21.9
CL (mL/h)	42.0	8.20	8.68	6.21	7.86
V_z (L)	11.7	5.0	4.1	3.6	6.0

Data are presented as arithmetic mean in each cohort. n, number of participants. $AUC_{0\text{-}t}$: area under the concentration-time curve from time 0 to the last quantifiable concentration; $AUC_{0\text{-}inf}$: AUC from time 0 to infinity; C_{max}: maximum serum concentration; T_{max}: time to maximum serum concentration; $t_{1/2}$: half-life; CL: total body clearance; V_z: Volume of distribution; λ_z: Terminal elimination rate

Supplementary Methods

Humanization of 8D6. The amino acid sequence of VH and Vκdomains from 8D6 were aligned with those of the human germline VH1-69/JH4 and Vκ1-39/Jκ1 alleles. To make the CDR graft, the acceptor VH framework, which differs from the human germline VH1-69 allele at 4 positions M48I, R66K, V67A, and S76N, was used. The CDRs, which are position 26-35 (CDR-H1), 50-56 (CDR-H2), and 93-104 (CDR-H3) of 8D6 were engineered into the acceptor VH framework to generate a direct CDR-graft of 8D6 VH as shown in the supplemental Fig. S1a. In the Vκ domain, the CDRs of positions 27-34 (CDR-L1), 50-56 (CDR-L2), and 89-97 (CDR-L3) were grafted to the acceptor Vκ framework, which differs from the human germline Vκ1-39 allele at a position M4L (Supplementary Figure 1B).

Binding to the FcεRI-bound IgE on ELISA. ELISA plates were coated with FcεRIα-Fc γ at 4°C overnight and blocked with assay diluents for 1 hour at RT. The plates were then saturated with 1 μg/mL human IgE. After washing with wash buffer, the captured IgE was incubated with UB-221 (hu8D6), c8D6, Omalizumab, and a murine anti-human IgE mAb 5H2 (AbD Serotec) at 10, 1, 0.1, and 0.01 μg/mL. The human IgG was detected using HRP-conjugated goat anti-human kappa light chain (GeneTex) and HRP-conjugated goat anti-murine IgG.Fc (Jackson ImmunoResearch). The color developed with TMB substrate solution was detected at 450 nm on the Molecular Devices microplate reader.

Binding to IgE on RBL SX-38 basophils. RBL SX-38 cells, rat basophilic leukemic cells transfacted with genes encoding the α, β, and g chains of human FcεRI were used as a pool of cell surface FcεRI. RBL SX-38 cells at 2x10^6/mL in FACS buffer were incubated with IgE at 3 μg/mL for 30 minutes on ice. After washing, cells of 2x10^5 cells in 100 ml FACS buffer were incubated with hu8D6, c8D6, Omalizumab, and 5H2 antibody at 10 μg/mL for 30 minutes on ice, followed by washing. Bound antibodies were detected by FITC-labeled goat IgG specific for human IgG-Fc or FITC-labeled F(ab)'2 rabbit anti-murine IgG (AbD Serotec). The stained cells were analyzed on FACS Canto II. Humanized 8D6 (UB-221), c8D6, and omalizumab could not bind to IgE-saturated RBL SX-38 cells, but 5H2 was bound to RBL SX-38 cells.

Binding of UB-211:IgE complex to and activation of rat huFcεRI$^+$-RBL SX-38 cells. RBL SX-38 at 3 x 10^5 cells were seeded in a 24-well plate in 0.5 mL of culture medium overnight in a 37°C incubator. On the next day, each well was washed twice with 0.5 mL of Tyrode's buffer and then 0.25 mL of pre-warmed Tyrode's buffer containing 10 μg/mL of the anti-IgE:IgE immune complexes (UB-221:IgE, c8D6:IgE, and omalizumab:IgE immune complexes at 1:1 molar ratio) or 1% Triton X-100 was added and incubated for 30 min at 37°C, the supernatants were collected and centrifuged at 300xg for 5 minutes at RT and 50

μL of clear supernatant were transferred from each well to that of a new 96-well black OptiPlate™ (Perkin-Elmer). 4-MUG substrate solution (50 μL) was added to each well and the plate was incubated at 37 °C for 1 hr. The reaction was terminated by adding 100 μL glycine buffer.

Binding of UB-221:IgE complex to $CD23^+$-SKW cells. SKW6.4 cells at $2x10^5$ in 100 μL of Ca^{2+}/Mg^{2+} FACS buffer were incubated with anti-IgE:IgE complexes (UB-221:IgE, c8D6:IgE, omalizumab:IgE at 1:1 molar ratio) and anti-CD23 antibody at 10 μg/mL for 30 minutes on ice, followed by washing with Ca^{2+}/Mg^{2+} FACS buffer. Bound anti-IgE:IgE immune complexes were detected by FITC-labeled goat IgG specific for human IgG-Fc or FITC-labeled $F(ab)'_2$ rabbit anti-murine IgG. The stained cells were analyzed on a FACS Canto™ II flow cytometer.

Binding of anti-IgE mAbs to CD23-bound IgE on ELISA. ELISA plates were coated with purified ILZ-CD23 at 5 μg/mL and blocked, and the coated ILZ-CD23 was then saturated with 3 μg/mL of purified human IgE in Ca^{2+}/Mg^{2+} assay diluents (1 mM $CaCl_2$, 0.5 mM $MgCl_2$, 0.5% BSA, 0.05% Tween-20, and 0.01% thimerosal in PBS). After washing with wash buffer, the captured IgE was then incubated with UB-221, c8D6, omalizumab, and murine anti-IgE mAb 5H2 at 10, 1, 0.1, and 0.01 μg/mL in Ca^{2+}/Mg^{2+} assay diluents. The bound human IgG and murine IgG were detected using HRP-conjugated goat anti-human IgG.Fc and HRP-conjugated goat anti-murine IgG.Fc. The color developed with TMB substrate solution was detected at 450 nm on the Molecular Devices microplate reader.

Competitive inhibition of IgE binding to FcεRI by UB-221 vs. ligelizumab on ELISA. Competitive inhibition (neutralization) by UB-221 vs. ligelizumab of IgE binding to FcεRI was investigated based on the free, unbound IgE that remained after incubation on 96-well ELISA plates. The plates were coated with FcεRIα-Fcγ overnight at 4 ^{o}C. UB-221 and ligelizumab, from three sources (two UBP lots, 040620 and 090320; and Creative Biolabs, CN: TAB755), were serially diluted at a range of 4 to 20,000 ng/mL and incubated with 200 ng/mL IgE at a 1:1 volume ratio for 1 hour at RT, and then the drug:IgE mixtures were added to the FcεRI-coated plates for 1-hour incubation at RT. After incubation and washing, the plate-bound IgE was detected by a biotin-conjugated mouse anti-human IgE antibody, followed by streptavidin-poly HRP. The color developed with TMB substrate solution was measured at 450 nm.

IgE binding affinity on SPR. The kinetic assay of IgE binding to UB-221, omalizumab, and ligelizumab was performed on SPR using the GE Healthcare Biacore X100 device. HBS-EP+ was used as a running buffer at a flow rate of 10 μL/min. Anti-human IgG Fc fragment antibodies were first covalently coupled in the CM5 sensor chip at pH 5.0 by standard amine coupling, and then the anti-IgE antibodies at 1 μg/mL were captured. Different concentrations

of human full-length IgE at 0.312-50 nM were injected for 120 seconds and the dissociation was measured for 720 seconds under constant buffer flow. The dissociation rate constant (Kd), association rate constant (Ka), and the equilibrium dissociation rate constant (K_D) were calculated using a 1:1 Langmuir curve fitting model.

RBL SX-38 degranulation assay. The rat basophilic hFcεRI$^+$-RBL SX-38 cells were co-incubated with 1 μg/mL of OVA-specific hIgE (AllerMAbs) and 0.002 to 15 μg/mL of UB-221 or omalizumab) for 2 hours at 37 °C. The cells were washed and degranulation reactions were induced with OVA (10 μg/ml OVA in 1% Triton X-100) for 30 minutes. The supernatant with cell-releasing β-hexosaminidase was harvested and incubated with citric acid-containing 4-MUG (4-methylumbelliferyl-*N*-acetyl-β-D-glucosaminide (Sigma-Aldrich) for 1 hour. The resulting fluorescence (excitation 355 nm; emission 460 nm) was measured for the calculation of % degranulation.

Competitive inhibition of IgE-binding to FcεRI on RBL SX-38 cells. FcεRI-expressing RBL SX-38 cells at 1×10^5 were mixed with 400 ng/mL of human IgE for 1 hour on ice. After washing with 1% BSA/PBS, UB-221 or omalizumab at 6 to 25,000 ng/mL were added into cells and incubated for 1 hour on ice. The cells were washed and incubated with 0.75 μg/mL of PE-conjugated anti-human IgE (eBioscience) for 30 min on ice. The stained cells were analyzed on the BD FACS Verse cytometer.

Effects of UB-221-vs.-omalizumab on IgE protein production in PBMCs and ELISA assays of total IgE, IgM, and IgA. Human peripheral blood mononuclear cells (PBMCs) from fresh whole blood of 11 healthy donors were isolated by Ficoll-Paque Plus (GE healthcare). In a 24-well plate, PBMCs at 1×10^6 cells/mL/well were cultured in complete IMDM (Iscove's Modified Dulbecco's Medium). The cells were stimulated with the anti-CD40 antibody (G28-5) (Biolegend) and IL-4 (R&D systems) at a final concentration of 100 ng/mL for 7 and 11 days at 37 °C, 5% CO2 in the presence of 1, 3, or 10 μg/mL of UB-221 or omalizumab. Untreated cells served as controls. The cell supernatants were assayed by ELISA for total IgE, IgM, and IgA.

For assay of total IgE (free plus mAb:IgE complexes) in supernatants from the IgE-PBMC production studies, ELISA plates (Nunc) were coated with each mAb at 0.7 μg/mL overnight at 4 °C. The plates were blocked with 0.2% N-Z-Amine B buffer for 1 h at room temperature (RT) and washed with PBS/0.05% Tween 20. The samples collected from UB-221-treated PBMCs and IgE calibration standards at 19.5 – 5000 ng/mL were spiked with UB-221 at 100 μg/mL for a particular dose group. Similarly, the IgE standards and samples treated with omalizumab or ligelizumab (UBP lot) were prepared in the same way. After incubation for 1 h at RT, IgE was detected with monoclonal mouse anti-human IgE Fc-HRP. The color developed with TMB substrate solution was measured at 450 nm on the Molecular Devices

microplate reader (SpetraMax M2e) with SoftMaxPro software. Total IgM and IgA in supernatants were assayed using Human IgM and IgA ELISA quantitation sets (Bethyl). ELISA plates were coated with 1 μg/mL of goat anti-human IgM or anti-IgA. Calibration standards were prepared in the culture medium at the range of 15.6 to 1,000 ng/mL for IgM and 7.8 to 5,000 ng/mL for IgA. The captured IgM and IgA were detected with HRP conjugated goat anti-human IgM and anti-IgA antibodies, respectively.

Binding of anti-IgE mAbs to the CD23-bound IgE. The 96-well ELISA plates were coated with 100 μL of CD23 at 5 μg/mL, and the next day, 100 μL of 100 ng/mL IgE was added and incubated for 1 hour at RT. After washing, serially diluted UB-221, omalizumab, or ligelizumab (UBP lot) at 0.0001 – 100 μg/mL were added and incubated at RT for 1 hour. The binding of the anti-IgE mAbs was detected with an HRP-conjugated goat anti-human IgG Fc (Jackson Immuno Research).

Binding of mAb:IgE complex to CD23. The binding of anti-IgE mAbs in IgE-complex form to the CD23 was investigated on CD23-immobilized ELISA. On day 1, 100 μL of human IgE at 8 μg/mL were mixed with 100 μL of UB-221, omalizumab or ligelizumab (UBP lot) at 0.00488 to 20 μg/mL (1:4 serial dilution), and 96-well ELISA plates were coated with 100 μL of 5 μg/mL CD23. The samples and plates were incubated at 4 ^{o}C overnight for 16 hours. On the next day, 100 μL IgE-drug mixtures prepared on the last day were added to the CD23-coated ELISA plates and incubated at RT for 1 hour. The binding of mAb:IgE complex to CD23 was detected with an HRP-conjugated goat anti-human IgG Fc (Jackson Immuno Research).

Free chimerized IgE in sera of hIGHE-knockin mice. In the genome of the hIGHE-knockin mouse, the Cγ1 constant region is replaced by a human Cε constant region. For determination of the free serum concentrations of free chimerized IgE in mice treated with a single i.p. dose of UB-221, FcεRIα-Fcγ at 1 μg/mL was immobilized on White Opaque 384-Well Microplates (Nunc) at cold overnight. The 10-fold diluted serum samples and the serially diluted IgE calibration standards at 3.13-200 ng/mL were added into plates and incubated at RT for 1 hour. For color development, the samples were treated with the biotinylated mouse anti-human IgE (BD Pharmingen) at 100 ng/mL and then with streptavidin-poly HRP (Thermo) at 1:40,000 dilution. After washing, plates were incubated with SuperSignal ELISA Pico Chemiluminescent substrate (Thermo, CN: 37070) for 10 min in the dark. Without stopping the reaction, the relative light units were measured at 425 nm.

Free IgE concentrations in sera of cynomolgus macaques after a single dose of UB-221. The serum free cynomolgus IgE (cIgE) after receiving a single i.v. dose of UB-221 was quantitated by ELISA coated with 100 μL/well of 0.5 μg/mL FcεRIα-Fcγ and incubated overnight at cold. Standards (2 to 1000 ng/mL of cyno IgE.Fc fragment), quality control

samples (25, 100, and 250 ng/mL), and serum samples (diluted in IgE-deleted diluent) were incubated on FcεRI-coated plates for 1 hour. After washing, the captured cyno IgE was detected by 100 μg/mL of biotin-conjugated mouse anti-human IgE antibody (Miltenyi Biotec, crossreact with cIgE) and s1:20,000 treptavidin-poly HRP (Thermo Pierce). The color developed with TMB substrate solution was measured at 450 nm. The lower limit and the upper limit of quantitation were 10 and 800 ng/mL, respectively.

Serum UB-221 concentrations in cynomolgus macaques after a single dose of UB-221. The serum UB-221 concentrations in cynomolgus macaques after receiving a single i.v. dose of 5.0 mg/kg UB-221 were quantitated by ELISA coated with 100 μg/mL of 2 μg/mL mouse anti-human IgG kappa mAb (Antibody Solutions) at cold overnight. Standards (UB-221 in a range of 0.98 to 500 ng/mL), quality control samples (5, 20, and 100 ng/mL), and serum samples (diluted in serum matrix) were incubated with the coated plates. After washing, the plate-bound UB-221 was detected by an HRP-conjugated mouse anti-human IgG Fc antibody (Southern Biotech), diluted 1:40,000. The color developed with TMB substrate solution was measured at 450 nm. The lower limit (LLOQ) and the upper limit of quantitation (ULOQ) were 0.98 and 500 ng/mL, respectively.

Free IgE remained in buffer solution after competitive inhibition of IgE binding to FcεRI. Competitive inhibition (neutralization) by UB-221 vs. omalizumab of IgE binding to FcεRI was investigated based on the free, unbound IgE that remained after incubation on 96-well ELISA plates. The plates were coated with FcεRIα-Fcγ overnight at 4 ^{o}C. UB-221 and omalizumab were serially diluted at a range of 4 to 20,000 ng/mL and incubated with 200 ng/mL IgE at a 1:1 volume ratio for 1 hour at RT, and then the drug:IgE mixtures were added to the FcεRI-coated plates for 1-hour incubation at RT. After incubation and washing, the plate-bound IgE was detected by a biotin-conjugated mouse anti-human IgE antibody, followed by streptavidin-poly HRP. The color developed with TMB substrate solution was measured at 450 nm.

Superposed 3D structures of mAb:IgE:CD23, mAb:IgE, and FcεRIα:IgE for anti-IgE mAbs. The 3D illustrations of the superposed mAb:IgE:CD23 complex structure are generated using the Protein Data Bank accession number 6EYO (8D6:IgE), 6UQR (ligelizumab:IgE), 5HYS (omalizumab:IgE), 4EZM (IgE:CD23), and 2Y7Q (FcεRIα:IgE). The 8D6 represents the murine parent mAb of UB-221. Superpositions of 3D structures were performed using both PyMOL and CCP4 Software Suite. The coordinates were superimposed on one another with SUPERPOSE in CCP4 Suite by superposing the Cε3 and Cε4 domains of IgE to generate the new coordinates. The aligned and transformed coordinates were superposed and structure presentations were generated using the PyMOL program. Interfacial areas were calculated with AREAIMOL in the CCP4 program suite. The contact areas for

each of mAb:IgE and FcεRIα:IgE complexes were calculated on the Van der Waals surface of an atom within 3.8 Å. The structure presentations were generated using the PyMOL program.

Apoptosis to mIgE-Ramos by anti-IgE mAbs. Apoptotic effects were evaluated using mIgE.FcL-expressing Ramos cells, which were incubated with increasing concentrations of UB-221, omalizumab, or ligelizumab (UBP lot) in RPMI 1640 medium for 1 h at 37˚C. The cells were then treated with the 10 μg/mL goat F(ab')2 (Jackson Immuno Research Laboratories, West Grove, PA) specific for Fc of human IgG (2nd Abs) for 24 hours at 37 ˚C. The extent of apoptosis of the cells was measured by staining cells with a staining solution containing PE-labelled annexin V (Southern Biotech) and 2.5 μg/mL propidium iodide (PI) for 15 min in the dark at RT. The stained cells were analyzed on a FACSVerse flow cytometer (BD Biosciences, San Jose, CA). The percentage of apoptotic cells, defined as annexin V-positive, was obtained in a dot plot analysis.

Antibody-dependent cellular cytotoxicity (ADCC) to CD23-expressing SKW6.4 cells. Peripheral blood mononuclear cells (PBMCs), as ADCC effector cells, were isolated from whole blood samples of four healthy donors (2 males and 2 females) by a density gradient centrifugation method using Ficoll-PagueTM PLUS (BD, CN. 17-1440-03). Responsive human B-lymphoma cell line SKW6.4, used as target cells, was labeled with CFSE (Carboxyfluorescein succinimidyl ester, eBioscience, CN. 65-0850-84) as target cells. SKW6.4 cells were washed once with 3 mL of 0.1% BSA/PBS and 3 x 10^6 cells were suspended in 3 mL of 0.1% BSA/PBS. 3 μL of 1 mM CFSE was added into cell suspension (final concentration of CFSE was 1 μM) and incubated for 10 min at 37°C. 10 minutes later, a cold complete medium was added to stop the labeling reaction. Then, cells were washed twice with a cold complete medium and CFSE-labeled SKW6.4 cells were prepared. ADCC activity was assessed by a flow-cytometry-based method. Target cells were incubated with human IgE at 1 μg/mL for 30 minutes at 37oC. Then, IgE-bearing target cells were incubated with GA101 (Gazyva, Roche), omalizumab (Xolair, Novartis), or UB-221 from concentrations ranging from 0.05 to 5000 ng/mL for 15 to 30 minutes at 37 ^{o}C, respectively.

Samples of 2x10^4 target cells were mixed with the effector cells at an effector to target ratio of 20:1 and incubated for 4 hours at 37°C. After 4 hours of ADCC reaction, 20 μL of 7-AAD (7-Aminoactinomycin D, eBioscience, CN. 00-6993-50) was added into samples to identify dead cells and incubated on ice for at least 15 minutes. CFSE-positive cells were gated to distinguish target cells from the effector/target mixture. CFSE positive cells were further analyzed for their 7-AAD signals. If cells had negative 7-AAD signals, they were considered as a live cell population. The percentages of live cells of total CFSE-positive cells with or without mAb treatment were used for ADCC calculation as described in the following formula:
%ADCC = 100x [(%of live target cells without mAb) – (%of live target cells with mAb)] /

[% of live target cells without mAb].

Complement-dependent cytotoxicity (CDC) to CD23-expressing SKW6.4 B-lymphoma cells. The CD23-overexpressed SKW6.4 B-lymphoma cells at 10^6 cells/mL were incubated and preabsorbed with 800 ng/mL human IgE for 30 min on ice. Excess IgE was washed out and the cell density was adjusted to $2x10^6$ cells/mL and 50 µL cell suspension was applied per well into a microplate, to which 50 µL RPMI-1640 medium containing 20% human serum and UB-221 at a concentration range of 0.078 to 10 µg/mL was added. The anti-CD20 rituximab was used as a positive control and omalizumab as a negative control. After incubated at 37 ^{o}C for 1 hour, 100 µL CellTiter-Glo 2.0 reagent (Promega, CN: G9242/3) was added to each well and incubated at RT for 10 minutes. The intensity of luminescence was determined by a microplate reader (Molecular Devices, SpetraMax M2). Medium with cells represents the maximal luminescence, and medium without cells represents the basal luminescence. The CDC% was calculated as:
% specific lysis = (Maximal RLU - Experimental RLU) / (Maximal RLU - Basal RLU) x 100%.

Measurement of UB-221 in sera of Phase-1 study participants. Serum concentrations of UB-221 were determined by a sandwich enzyme linked immunosorbent assay (ELISA). Briefly, 96-well MaxiSorp microplate (Nunc) were coated with 1 µg/mL idiotypic anti-UB-221 antibody (in-house preparation) at 4℃ overnight. Serum samples were first diluted 200-folds, 5,000-folds or 125,000-folds in diluent (0.5% CHAPS, 30 mM NaCl and 5% BSA and in 1X PBST) according to the expected UB-221 concentration. After blocking the plate with 5% BSA (AppliChem) in 1X PBST (Thermo), the diluted serum samples and serially diluted UB-221 calibration standards at 0.098-100 ng/mL were then loaded to the coated plate and incubated at RT for 1 hour. After washing with 1X PBST, the wells were loaded with 100,000-fold diluted mouse anti-human IgG Fc-HRP antibody (H2, Southern Biotech) and incubated at RT for 1 hour. After washing with 1X PBST, OptEIA TMB substrates (BD Bioscience) were added and incubated 15 minutes for signal development and stop with 2N sulfuric acid (Sigma). The signals were measured with OD_{450} values using SpectraMax M2e microplate reader (Molecular Devices) and the concentrations of UB-221 were determined by interpolation from the calibration curve fitted with 4-parameter logistic regression using SoftMax Pro GxP software 7.0.3 (Molecular Devices). The detection limits of UB-221 was 80 ng/mL.

Measurement of free IgE in sera of Phase-1 study participants. Serum concentrations of UB-221-free IgE were determined by a sandwich enzyme linked immunosorbent assay (ELISA). Briefly, 96-well MaxiSorp microplate (Nunc) were coated with 2 µg/mL

recombinant protein, FcεRIα-IgG.Fc (in-house preparation), at 4℃ overnight. Serum samples were first diluted 4-folds or 250-folds in diluent (5mM EDTA, 0.5% CHAPS, 30mM NaCl and 1% BSA in 1X PBST) according to the expected free IgE level in serum. After blocking the plate with 1% BSA (AppliChem) in 1X PBST (Thermo), the diluted serum samples and serially diluted IgE-spiked calibration standards at 3.125-200 ng/mL were then loaded to coated plate. After washing with 1X PBST, the wells were first incubated with 10,000-folds diluted biotin-conjugated monoclonal mouse anti-human IgE antibody (G7-26, BD Bioscience) for 1 hour and then 15,000-folds diluted streptavidin poly-HRP (Thermo) for another hour at RT. After washing with 1X PBST, OptEIA TMB substrates (BD Bioscience) were added and incubated 15 minutes for signal development and stop with 2N sulfuric acid (Sigma). The signals were then measured with OD_{450} values using SpectraMax M2e microplate reader (Molecular Devices) and the concentrations of UB-221-free IgE were determined by interpolation from the calibration curve fitted with 4-parameter logistic regression using SoftMax Pro GxP software 7.0.3 (Molecular Devices). The detection limits of UB-221-free IgE was 24 ng/mL.

Measurement of FcεRI expression on basophils of Phase-1 study participants. Peripheral blood samples were collected with heparin vacutainer and processed for determining FcεRI expression on basophil at approximately 24 hours after sample collection. Whole blood was first incubated on ice for 30 minutes with the following fluorescence-labeled antibodies: FcεRIα-FITC (AER-37), CD193(CCR3)-PE (5E8), and CD3-PerCP (HIT-3a) (all from BioLegend). After staining, the samples were lysed and fixed with 1X RBC lysis/fixation solution (BioLegend) for 20 minutes in the dark and washed twice with 1X PBS (Gibco). After washing, the samples were then acquired on BD FACSVerse flow cytometer (calibrated with CS&T research beads daily) with BD FACSuite software (all from BD Biosciences). The data were analyzed using FlowJo software version 10.8 (FlowJo LLC). Briefly, a large gate was first drawn that include both lymphocytes and monocytes on the FSC/SSC. Basophils were then defined as $CD193^{+}CD3^{-}$ population in the gate. The FcεRI expression level was then determined by mean fluorescence intensity (MFI) of FITC in basophil population.

Formation of UB-221:IgE immune complexes in PBS and human serum by FDS-AUC. Alexa-UB-221 mAb and human IgE were mixed at a different molar ratio in PBS or human serum and incubated at 20 °C for approximately 2 hours. Alexa-mAb:IgE complexes formed at 10:1, 5:1, 3:1, 1:1, 1:3, 1:5, and 1:10 molar ratio characterized by FDS-AUC FDS-AUC experiments were performed at 20 °C using fluorescence optics (Aviv Biomedical) at a rotor speed of 40,000 rpm. Data were acquired at 20-µm radial increments and averaging 5 revolutions/scan. To prevent non-specific adsorption of protein to centerpiece walls and

windows, 0.1 mg/mL lysozyme (Sigma Aldrich, CAT 4919) was added to PBS. Because Alexa-mAb is formulated in PBS, 250 nM Alexa-mAb sample contained 80% (v/v) serum and 20% (v/v) PBS. To make solvent conditions uniform, all “serum” samples were prepared in 80% (v/v) serum.

Data acquired in PBS were analyzed with SEDFIT (version 14.6e) using the sedimentation coefficient distributions c(*s*) with a 68% confidence level. Data recorded for serum samples were analyzed using the c(*s*) method with the signal magnification radial-gradient option3. Fitted curves matched the shape of the experimental data with considerable accuracy, and the resulting root mean-square deviations (rmsd) did not exceed 1% of the total loading signal, indicating that good fits to the experimental data were achieved. The partial specific volume of mAb was calculated from the amino acid composition using the program SEDNTERP 1.09 and was 0.7261 cm^3/g. The apparent *s*-values were converted to *s20,W* using density (1.00518 for PBS; 1.01797 for 80% serum) and viscosity (1.021 for PBS; 1.459 for serum) measured using an Anton Paar density meter DMA4500 and viscometer Lovis 2000ME, respectively.

Binding of anti-IgE mAbs to mIgE-Ramos B-lymphoma cells. Direct binding of anti-IgE mAbs to the IgE-bearing Ramos B cells was investigated on flow cytometry. Aliquots of 100 μL mIgE-Ramos B-lymphoma cells at 5×10^6 cell/mL were incubated with 100 μL of UB-221, omalizumab, or ligelizumab (UBP lot) at 0.0048 to 2.0 μg/mL in a staining buffer (2% FBS, 2 mM EDTA in PBS) for 1 hour on ice. After washing, 5 μL of PerCP-cy5.5 conjugated anti-human IgG (Biolegend) was added in 100 μL of cells resuspended in flow staining buffer and incubated on ice for 30 min. Flow cytometry was performed on the BD FACSVerse device.

Supplementary Appendix

Appendix 4

Phase 1 Study A-107 protocol

Appendix 5

Phase 1 Study A-107 IRB approval letter

Appendix 6

Phase 1 Study A-107 Informed Consent Form (ICF)
